# Increasing engagement with cognitive-behavioral therapy (CBT) using generative AI: a randomized controlled trial (RCT)

**DOI:** 10.1038/s43856-025-01321-8

**Published:** 2026-01-15

**Authors:** Jessica McFadyen, Johanna Habicht, Larisa-Maria Dina, Ross Harper, Tobias U. Hauser, Max Rollwage

**Affiliations:** 1 Limbic Limited, London, UK; 2 Department of Psychology, Institute of Psychiatry, Psychology & Neuroscience, King’s College London, London, UK; 3 Max Planck UCL Centre for Computational Psychiatry and Ageing Research, University College London, London, UK; 4 Department of Psychiatry and Psychotherapy, Medical School and University Hospital, Eberhard Karls University of Tubingen, Tübingen, Germany; 5 German Center for Mental Health (DZPG), Tübingen, Germany

**Keywords:** Anxiety, Depression

## Abstract

**Background:**

Shortages in mental healthcare lead to long periods of inadequate support for many patients. While digital interventions offer a scalable solution to this unmet clinical need, patient engagement remains a key challenge. Generative artificial intelligence (genAI) presents an opportunity to deliver highly engaging, personalized mental health treatment at scale.

**Methods:**

In a pre-registered (ClinicalTrials.gov: NCT06459128, 10 June 2024), parallel, 2-arm, unblinded, randomized controlled trial (N = 540), we evaluate whether a genAI-enabled cognitive behavioral therapy (CBT) app enhances engagement or symptom reduction compared with digital CBT workbooks. Eligible participants are adults residing in the United States with elevated self-reported symptoms of anxiety (GAD-7 ≥ 7) or depression (PHQ-9 ≥ 9), recruited online. After an online baseline assessment, participants are automatically randomly allocated (3:2) to receive either the genAI-enabled app or a digital workbook, both self-guided over six weeks. Primary outcomes are: 1) engagement frequency and duration, and 2) change in anxiety (GAD-7) and depression (PHQ-9) symptom severity. Secondary outcomes include adverse events and functional impairment. The study is unblinded to participants and researchers due to the nature of the digital interventions.

**Results:**

A total of 540 participants are recruited and randomized to each group (intervention: n = 322, active control: n = 218). Nine participants from the control group are excluded from analysis due to protocol deviations. Over six weeks, the genAI solution (n = 322) increases engagement frequency (2.4×) and duration (3.8×) compared to digital workbooks (n = 209), with moderate to large effect sizes. We observe comparable outcomes for anxiety (GAD-7) and depression (PHQ-9) with no differences in adverse events. Moreover, exploratory analyses suggest that participants who choose to engage with clinical personalization features powered by genAI experience stronger anxiety symptom reduction and improved overall wellbeing.

**Conclusions:**

Our findings suggest that, in self-directed usage, tailored genAI-enabled therapy safely enhances user engagement above and beyond static materials, without showing an overall enhancement in anxiety or depression symptom reduction.

## Plain language summary

Access to mental health care is often limited, leaving many people without support while they wait for treatment to start or between therapy sessions. Self-help tools can help fill these gaps but users often struggle to stay engaged. Generative artificial intelligence (AI), a technology that can generate new content like text or images, could make these tools feel more personal and interactive. In this six-week randomized-controlled trial with 540 adults experiencing anxiety or depression symptoms, we compared an AI-enabled cognitive-behavioral therapy (CBT) app with digital workbooks. People using the AI app engaged more often and for longer, while safety and symptom reduction were similar across groups. Those who used the app’s more personalized features showed the greatest improvements, suggesting AI-powered therapy tools could safely help people stay engaged between therapy sessions.

## Abstract (web summary)

McFadyen et al. test whether a generative AI-powered app could improve engagement with self-help cognitive behavioral therapy compared with digital workbooks. The AI app was used more often and for longer, with similar effectiveness, suggesting it can enhance engagement in digital mental health care.

## Introduction

Prolonged gaps in mental health care – whether while waiting for treatment to begin or in-between therapy sessions – leaves patients without adequate support, increasing the risk of symptom worsening, treatment drop-out, and adverse outcomes^1–3^. Addressing these critical gaps with timely and engaging interventions is essential for improving patient outcomes^1^. For cognitive-behavioral therapy (CBT), meaningful engagement with therapeutic materials outside of sessions, often in the form of structured “homework,” is a key predictor of improved clinical outcomes and therapy adherence^4^. Homework encourages patients to incorporate what they have learned from therapy into their everyday life, reinforcing and generalizing new skills, thereby promoting behavioral and cognitive change that results in better treatment outcomes^5^.

Limited availability of clinical staff and the high costs of continuous human supervision substantially limits the availability of labor-intensive solutions to fill these gaps, such as crisis hotlines, teletherapy, or messaging services^6^. Some approaches, such as guided or non-guided internet CBT (iCBT) and blended therapy, attempt to address shortages of clinicians by providing tools such as self-help workbooks or content delivered digitally through apps or online platforms. However, while both guided and non-guided engagement with iCBT tools can be clinically effective^7,8^, they typically present generic, “one-size-fits-all” content solutions that often struggle to engage patients^9^ due to a lack of crucial elements like interactivity and personalization that help patients make meaningful progress^10–12^.

The advent of large language models (LLMs) presents a transformative opportunity to overcome these limitations. Unlike traditional digital interventions, LLM-based generative AI (genAI) can facilitate highly interactive and personalized experiences that closely mimic therapist-patient interactions^13,14^. By providing dynamic, responsive, and tailored support, LLM-powered applications can adapt in real-time to each user’s unique context, effectively bridging the gap when a human clinician is not available^15,16^. This level of personalization and engagement is unique to genAI and is not attainable with digital interventions. GenAI-enabled solutions therefore offer a critical advancement in enhancing patient engagement and clinical outcomes^17^. However, the use of LLMs in mental healthcare raises important safety considerations, particularly regarding the risks of AI hallucinations (generating false or misleading information) and potentially harmful responses to vulnerable users^18,19^. Addressing these risks requires robust clinical safety frameworks and careful system design.

To address challenges with therapeutic engagement, we leveraged these recent innovations in genAI and developed a clinically validated, genAI-powered smartphone application (“app”) called Limbic Care (https://www.limbic.ai/care). This app features a conversational chatbot designed to deliver personalized CBT interventions and psychoeducation and also to provide empathetic, non-interventional emotional support. This genAI-enabled app is powered by proprietary clinical AI – a sophisticated orchestration of LLMs and domain-specific machine learning (ML) models designed to ensure the safety, validity, and efficacy of patient-AI interactions^20^. This unique implementation of genAI aims to provide a personalized, user-centric experience that cultivates a relationship between the user and the application, which has the potential to increase engagement both in terms of quantity (e.g., how often the tool is used and for how long) and quality (e.g., enabling more meaningful interactions with material tailored to the user’s personal problems).

Here, we conducted a two-arm, parallel-group, unblinded, randomized controlled trial (RCT) with the goal of evaluating a genAI-powered CBT app (Limbic Care) against a common form of self-directed care (digital workbooks) on dimensions of engagement, safety, and symptom reduction. For the active control, we delivered static CBT content via a digital workbook (i.e., a PDF), such as that typically provided as homework or as a low intensity intervention in care systems like the UK’s National Health Service (NHS) Talking Therapies program. Our target population was adults with elevated anxiety and/or depression symptoms who could benefit from self-directed therapeutic support. Participants were not currently waiting for or undergoing therapy for their mental health, allowing us to evaluate the app’s effectiveness as a standalone digital intervention without human clinical input. This context reflects real-world scenarios where individuals might benefit from therapy but are either unable to access, waiting for, or not currently seeking traditional human-led therapy.

Our primary objectives were to assess: (i) whether the genAI-enabled app increased participant engagement with therapeutic activities, and (ii) its effectiveness in reducing symptoms of anxiety, depression, and sleep disturbances, and improving overall well-being in symptomatic individuals. Our secondary objectives were to assess the safety profile of the intervention in comparison to static self-directed care materials. We hypothesized that the genAI-powered app would be superior to the active control condition in reducing symptoms (primary outcome) and safety (secondary outcome), while providing the additional benefit of enhanced user engagement (primary outcome) through its highly interactive and personalized features.

In this six-week trial, we provided either Limbic Care or a digital workbook (randomized 3:2 allocation) to participants recruited from the general public and screened for anxiety or depression symptoms above a clinical threshold. Both tools provided psychoeducation and structured CBT interventions designed for a problem of the participants’ choice (low mood, worry, or sleep problems). The digital workbook presented this content through static text and images on a website, while the app presented the interventions through interactive dialogue enabled by genAI that tailored the intervention delivery to the user’s specific problems. The app also provided open-ended conversation as a means of emotional support, as well as “guided sessions” that embedded intervention delivery within a problem exploration framework, with clinically-guided intervention selection using AI.

Overall, we found that the genAI app increased engagement with therapeutic materials, with 2.4 times more frequent open rates and 3.8 times longer engagement duration, compared to digital workbooks. Both groups showed comparable reduction in anxiety and depression symptoms, with equivalent safety profiles. Exploratory analyses suggested that participants who chose to engage with the app’s AI-powered conversational therapy sessions (“guided sessions”) showed enhanced symptom reduction, generating hypotheses for further investigation.

## Methods

### Study design

This was a six-week, two-arm, unblinded, parallel-group RCT comparing the effectiveness of a genAI-enabled digital CBT app (Limbic) in delivering CBT exercises to a static digital workbook with the same CBT curriculum. The study was conducted as an open-label trial due to the nature of the interventions. Participants were randomly allocated to the intervention group (Limbic app) or active control group (PDF format) in a 3:2 ratio. This unequal allocation was chosen to enable more detailed analysis of app-specific engagement patterns and potential behavioral moderators while maintaining adequate statistical power for primary outcome comparisons. Ethical approval was obtained from the University College London (UCL) Research Ethics Committee [6218/003] on the 7th of May, 2024. The study design, including primary and secondary and additional outcome measures and their analyses, was registered on the ClinicalTrials.gov registry (NCT06459128) on 10 June 2024. All procedures were conducted in accordance with the Declaration of Helsinki.

### Participants

As a feasibility and preliminary efficacy trial, this study focused on establishing initial clinical efficacy, engagement, and safety in a non-clinical population with elevated symptoms. Eligible participants were United States residents aged 18+ years with anxiety and/or depression symptoms above threshold scores on widely validated screening measures. These pre-registered thresholds were defined using the Generalized Anxiety Disorder 7-item questionnaire^21^ (GAD-7 scores ≥ 8) and the Patient Health Questionnaire^22^ (PHQ-9; scores ≥ 10), respectively. These thresholds were selected to align with standard screening practices in primary care and psychological services, such as those used by the UK’s NHS Talking Therapies program, to identify individuals experiencing significant psychological distress who may benefit from intervention. Due to a protocol implementation discrepancy, the actual thresholds used in the study (GAD-7 ≥ 7 and PHQ-9 ≥ 9) were one point lower than the pre-registered thresholds. This minor deviation did not affect the clinical relevance of our sample, as both sets of thresholds fall within the established ranges for identifying meaningful symptomatology (GAD-7 and PHQ-9 scores ≥ 5 indicate mild to severe symptoms). These measures, while not diagnostic tools, are widely used and validated screening instruments in both clinical practice and research settings for identifying individuals with symptoms of anxiety and depression.

Inclusion criteria included fluency in English, access to a smartphone, and not currently receiving psychological therapy from a mental health professional. Exclusion criteria included high alcohol intake (≥ 10 alcohol units per week), frequent recreational drug use (more than weekly; specific drugs unspecified), recent changes in dosage or type of prescription medication for mental health (in the last 8 weeks), previous use of the Limbic app, and self-reporting being at risk of self-harm or causing harm to others. All participants were recruited online via the Prolific platform (https://www.prolific.com/) and provided informed consent. A target N of 540 was set based on a power calculation from observational patient data, designed to provide 90% power to detect a fixed effect of the group × week interaction in our linear mixed effects models (see Supplementary information).

### Intervention

#### Intervention: Limbic care

**Fig. 1:**
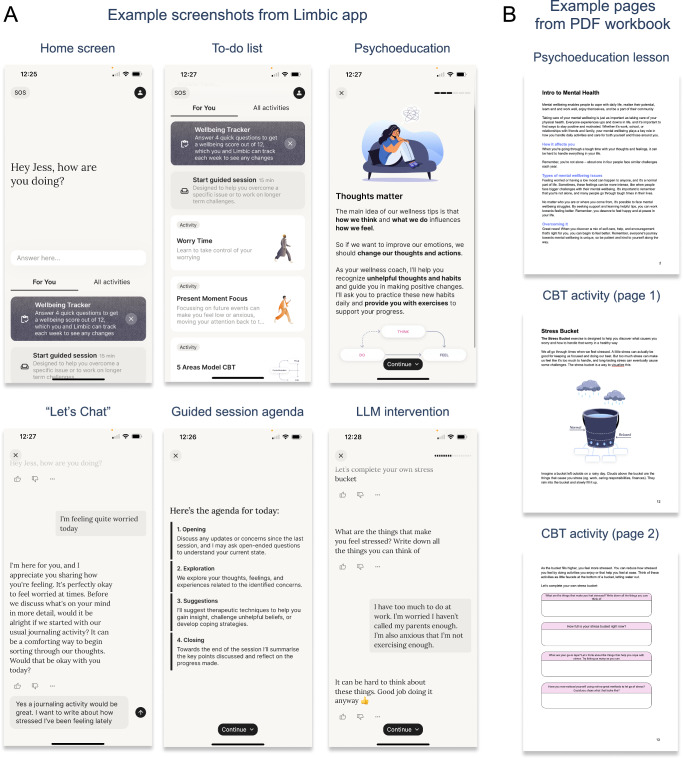
Wellbeing materials provided for the intervention (app) and control (digital workbook) groups. **A** Example screenshots from the Limbic app, featuring (from left to right) the home screen with the “Let’s Chat” feature at the top, followed by the “to-do list” of available psychoeducation lessons and CBT activities, with examples for a Let’s Chat conversation, guided session agenda, and CBT activity (or “intervention”) presented via a conversational interface using an LLM. **B** Example pages from the digital workbook used as an active control modelled off CBT worksheets standardly used in mental health treatment, with psychoeducation lessons and CBT activities presented as text and images.

Limbic Care (see Fig. 1; https://limbic.ai/care) is an app designed to deliver therapeutic content and mental health support. The app is designed to support adults (18 years and older) outside of traditional in-person therapy sessions. It can be used in-between therapy sessions, while waiting to start therapy, or as a standalone tool without therapist involvement. The intervention is centered around an AI-powered conversational chatbot that uses LLMs and clinically-specialized ML algorithms to assist users in completing therapeutic exercises (psychoeducation lessons and CBT activities), provide emotional support (through the “Let’s Chat” feature), and guide users through structured problem exploration sessions and clinically-personalized exercise suggestions (referred to as “guided sessions”). The primary aim of the app is to facilitate self-directed engagement with therapeutic materials, enabling patients to independently apply clinically-validated techniques in their daily lives. The app therefore serves to enhance accessibility and practical application of CBT principles.

Limbic Care’s genAI features utilize a sophisticated AI architecture that combines LLMs with a safety and clinical governance system referred to as a “cognitive layer” architecture. This architecture provides as an intelligent intermediary between users and the LLM, incorporating specialized machine learning classifiers and clinical logic to ensure safe and therapeutically appropriate interactions^20^. The cognitive layer architecture operates through a two-way filtering mechanism: it processes all user input to detect clinically-relevant information (such as crisis signals or specific therapeutic queries) and modifies LLM prompts accordingly, while also monitoring and validating LLM outputs to ensure clinical appropriateness. For instance, when users request specific therapeutic information, the system automatically retrieves validated content from a curated knowledge base rather than relying on LLM-generated responses. Similarly, if crisis signals are detected, the system redirects users to appropriate crisis support resources.

The app offers three main functionalities:

1. **“Let’s Chat” for Emotional Support**: The “Let’s Chat” feature enables users to engage in free-flowing conversation about their emotions and challenges. The underlying LLM, guided by carefully constructed clinical prompts and continuously monitored by the cognitive layer’s safety protocols, provides active listening and empathetic responses following person-centered therapy principles. This creates a supportive environment for users while maintaining clinical safety and appropriateness.
2. **Delivery of CBT Materials and Exercises (Activities and Psychoeducation)**: Users access clinically-validated CBT content through conversational interactions. The cognitive layer architecture ensures accurate delivery of:
  *Psychoeducation*: Short, digestible content focused on educating users about specific psychological concepts or coping strategies (e.g., understanding cognitive distortions, building resilience). These are either presented as “slides” containing text and images that a user can swipe through or can be delivered via the app’s conversational interface by querying the system’s validated knowledge base.
  *CBT Activities*: Structured exercises inspired by CBT principles – such as thought records, behavioral activation tasks, and mindfulness practices – delivered through interactive, personalized dialogue. These exercises are designed to help users actively work through negative thoughts and maladaptive behaviors.
3. **Guided Sessions**: This feature combines LLM-driven conversational capabilities with specialized clinical ML algorithms to deliver structured therapeutic experiences similar to human-led therapy sessions. The cognitive layer architecture implements a specialized clinical workflow that guides users through a process typical of CBT. During these sessions, the cognitive layer’s internally-validated machine learning classifiers analyze the conversation to identify clinically-relevant user states^20^. Based on this analysis, the system recommends specific CBT exercises that are most appropriate for addressing the identified patterns and states. This structured workflow ensures that each guided session follows therapeutic best practices while providing personalized support based on real-time analysis of the user’s psychological state. The clinical accuracy of this technology is detailed in prior work^20^, where it has been shown to enable off-the-shelf LLMs to perform at a standard comparable to, or exceeding, human clinicians on key text-based assessment benchmarks.

All interventions are delivered via an integrated interface that combines the conversational AI component with a therapeutic intervention to-do list, allowing patients to track their progress and complete assigned tasks systematically. In this study, participants’ to-do lists were populated on a predefined schedule with content that aligned with the “course” the participant selected. Three courses were offered, each targeting specific psychological concerns for managing sleep problems, worry, or low mood. The order of each course’s psychoeducation lessons and CBT activities was matched with the control condition.

#### Control: digital workbook

Participants in the control group received access to a digital CBT workbook developed specifically for this study to serve as an active control. The workbook’s content was developed and reviewed by a team of in-house certified CBT therapists, and its structure was closely modeled on evidence-based digital materials standardly used within the UK’s NHS Talking Therapies services, a large-scale public program for delivering psychological therapies.

The primary rationale for this in-house development was to ensure that the core CBT curriculum was matched across both arms. This design allowed for a direct comparison of the delivery mechanisms: the genAI intervention delivered the curriculum through dynamic, interactive dialogue, while the control condition delivered the same curriculum via a static, text-based workbook. This contrast allowed us to isolate the effects of the genAI-powered delivery itself (see Table 1 for a detailed comparison of features).

**Table 1:** Comparison of intervention and control features

Feature/Component	Active Control (Digital Workbook)	Intervention (Limbic Care App)
**Core CBT Curriculum**		
Psychoeducation materials	✓	✓
CBT exercise structure	✓	✓
Course structure (worry/mood/sleep)	✓	✓
**Delivery**		
Digital access	✓	✓
Smartphone compatibility	✓	✓
Progress tracking	×	✓
**GenAI Features**		
Conversational emotional support (“Let’s Chat”)	×	✓
Conversational learning support (querying Limbic’s database)	×	✓
Conversational exercise delivery	×	✓
Personalized guided sessions	×	✓

Just as in the app intervention, participants could choose from one of three courses with content tailored to managing sleep problems, worry, or low mood. Each workbook contained a combination of psychoeducational material and CBT intervention worksheets derived from the same curriculum as the intervention arm. These materials were presented as static documents, consisting of text, images, and blank response boxes. Participants could view their digital workbook on a smartphone, tablet, or computer, and could also print it out. The digital workbook therefore served as an active comparator representing a common delivery mode of self-directed therapy and CBT homework within healthcare settings.

### Procedure

Participants were recruited on Prolific and completed a screening questionnaire. Demographic information was retrieved from Prolific’s prescreening database. Eligible participants (see Participants section for inclusion/exclusion criteria) were invited to participate in the full study several days later, on the 5th of June 2024. Upon entering the study, participants first completed all baseline assessments, including the GAD-7 and PHQ-9. Only after completing these baseline measures were participants automatically randomized to either the intervention or control condition using Gorilla’s randomization algorithm^23^. This sequential procedure ensured that neither participants nor researchers could know or influence group allocation during baseline assessment. Randomization was not stratified by any baseline variables.

Following randomization, participants in the intervention condition received instructions for installing and signing in to the Limbic app on their smartphone, while those in the control condition received a URL to access the digital workbook of their choice (a course on sleep problems, worry, or low mood). Both interventions required participants to sign in with a unique participant identifier, allowing us to match engagement data with survey data.

Over a period of 6 weeks (ending on the 17th of July 2024), participants were invited to participate in a weekly survey containing questions about mental health, app/workbook engagement, and safety, as well as quantitative and qualitative measures of user experience and feedback (see Outcome measures). Thus, participants engaged in a 6-week intervention period, resembling typical waitlist durations or courses of CBT. Participants were compensated at a rate of £9/hr for completing the surveys and were awarded a £3 bonus at the end of the study if they completed all 6 weekly surveys. Critically, participants were not financially compensated for their engagement with either the Limbic app or digital workbook. They were encouraged to engage with their assigned materials 4 times per week but were clearly instructed that their engagement would not affect their payment.

### Outcome measures

#### Primary outcomes

Our primary outcome measures included: 1) engagement with the therapeutic materials, and 2) change in anxiety and depression symptom severity.

Engagement was operationalized through objective measures that were passively collected via built-in tracking functionality in both the Limbic app and the host website for the digital workbook. These objective measures included the total time spent in the app/viewing the workbook (engagement duration) and the total number of times these resources were accessed (engagement frequency). Both measures were summed per week and analyzed over time, as well as summed across the full 6-week intervention period. Additionally, participants provided subjective ratings of their engagement through weekly surveys, reporting their usage frequency (0 to 5+ times) and duration (<5 mins, 5-10 mins, 10+ minutes) on Likert scales, which were examined in exploratory analyses.

For symptom severity, we measured anxiety (GAD-7) and depression (PHQ-9) at baseline and then at weekly intervals throughout the 6-week intervention period. Both the GAD-7 and PHQ-9 scales are validated self-report scales used widely in healthcare settings as markers of mental illness^21,22^. Items are rated on a 4-point Likert scale ranging from 0 (“Not at all”) to 3 (“Nearly every day”), resulting in a total score between 0 and 21 (for GAD-7) or 27 (for PHQ-9). Scores on each scale were collected each week and analyzed from baseline to the end of the intervention period, with the total change from baseline to week 6 per scale used as the overall outcome measure.

#### Secondary outcomes

Following standard clinical trial safety procedures, we monitored all adverse events reported by participants throughout the study period. In each weekly survey, participants were asked: “Have you experienced any new adverse physical or mental health events in the past week?” Participants who responded “yes” were asked to describe the event. These descriptions were reviewed weekly by clinical researchers to identify any events meeting ISO 14155:2020 criteria for Serious Adverse Events (SAEs) so that the participant could be directed towards crisis support. We calculated both the total number of events reported per participant and the proportion of participants reporting any event across the 6-week study period.

#### Additional measures

##### Work and Social Adjustment Scale (WSAS)

Functional impairment was assessed using the Work and Social Adjustment Scale (WSAS), a 5-item self-report measure evaluating the impact of mental health symptoms on daily functioning across work, home management, social activities, and close relationships^4^. Each item is scored from 0 to 8, yielding a total score range of 0 to 40, with higher scores indicating greater impairment. Change in WSAS scores was analyzed from baseline to week 6, with weekly measurements.

##### Mini Sleep Questionnaire (MSQ)

Sleep quality and disturbances were evaluated using the Mini Sleep Questionnaire (MSQ), a 10-item self-report measure assessing excessive daytime sleepiness and sleep disturbances^24^. Each item is rated on a 7-point scale, with higher scores indicating greater sleep impairment. Change in MSQ scores was analyzed from baseline to week 6, with weekly measurements.

##### User experience

Custom rating scales and free-text responses were included in the baseline and weekly questionnaires to gauge participants’ satisfaction, acceptability, and perceived effectiveness of the allocated digital tool. Each item was rated on a 7-point Likert scale, with higher scores indicating more positive user experience. Measures included ease of use (“How easy was it to navigate the app/workbook?”), usefulness (“How useful did you find the app/workbook for your mental health?”), and motivation (“How motivated were you to use the app/workbook to improve your mental health?”). A full list of items can be found in the Supplementary information.

##### Beliefs and attitudes towards AI & psychotherapy

Custom rating scales and free-text responses were included in the baseline and final questionnaire in Week 6 to measure changes in beliefs and attitudes relating to digital mental health support tools. These included pre- and post-measures of trust in AI-enabled mental health tools (“In general, how much do you trust wellbeing apps that use artificial intelligence (AI)?”), preferences for workbooks vs apps for mental health support (“Imagine a mental wellbeing course was offered to you in the form of a [digital workbook (i.e., a PDF you could open on your computer or print out)]/[app that included an AI chatbot and interactive activities]. Which format would you prefer?”), and interest in pursuing therapy from a mental health professional (“How likely are you to arrange to see a therapist in the next 3 months?”).

##### Demographics

Demographic information including age, biological sex at birth, sexuality, education level, employment status, student status, and ethnicity were collected from Prolific’s prescreening database for all participants. Additional background information was collected in the baseline survey, including prior experience with therapy (experience with CBT, prior diagnosis), psychiatric medication status, how comfortable they feel with using digital tools, accessibility issues (e.g., deafness, blindness, etc.), and previous experience with mental wellbeing apps.

### Protocol amendments

**Fig. 2:**
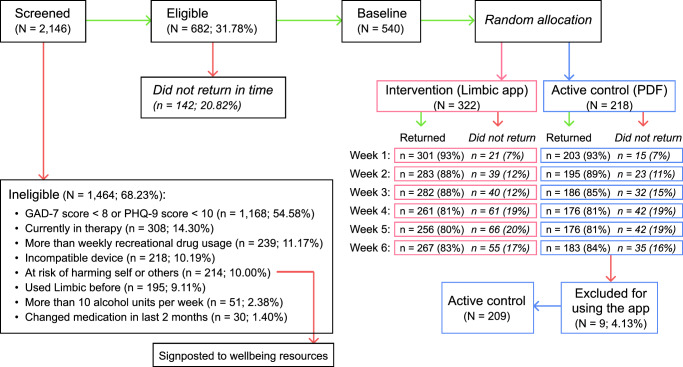
CONSORT diagram for the randomized controlled trial (RCT). GAD-7 is the Generalized Anxiety Disorder 7-item scale and PHQ-9 is the Patient Health Questionnaire 9-item scale. PDF refers to the file format of the digital workbook.

Three amendments were made to the pre-registered study protocol. First, while our study protocol specified a comparison of study completers between groups to assess potential attrition bias, we did not conduct this analysis as it was recognized that post-randomization dropout may be differentially affected by the interventions themselves. Instead, we focused on transparent reporting of attrition rates in our CONSORT flow diagram (see Fig. 2) and used appropriate methods for handling missing data in our primary analyses (see below).

Second, although the study was initially conceived as a non-inferiority trial in the protocol, our statistical analysis framework was ultimately conducted as a superiority trial to directly examine differences between the intervention and control groups. This approach was applied consistently across all primary, secondary, and additional outcomes.

Third, we updated our pre-registered primary outcome metric from “therapy completion” to “engagement duration”. This change was necessitated by technical limitations in our ability to track page-specific interactions within the PDF control condition, which prevented us from inferring which exercises had been viewed and thus likely completed. The updated metric of engagement duration presented a more reliable and more comparable metric between groups.

As specified in the protocol, an interim analysis was conducted at the trial midpoint for safety and futility monitoring. This analysis did not identify any concerns requiring protocol modifications or early trial termination.

### Power calculation

To estimate the required sample size for this study, we analyzed data from N = 240 patients enrolled in 2023 in cognitive behavioral therapy at an NHS Talking Therapies service that offered Limbic Care. These patients had above-threshold anxiety and/or depression symptoms (≥8 on GAD-7 or ≥10 on PHQ-9) in their initial assessment and had completed at least 2 treatment appointments. Only closed cases (i.e., treatment completed, patient dropped out, or patient referred to another treatment or provider) were analyzed.

Within this clinical dataset, we selected the total change in anxiety symptoms (GAD-7 difference from beginning to end of treatment) as a representative outcome for the four clinical outcome measures in the present study (GAD-7, PHQ-9, WSAS, and MSQ). We also selected the proportion of “did-not-attend” (DNA) appointments as a proxy for our treatment engagement outcome measures (see Table 2).

**Table 2:** Data and results for statistical power calculation for symptom reduction (approximated from known GAD-7 changes) and engagement (approximated from known changes in treatment engagement, as the “did-not-attend” – or “DNA” – rate)

Outcome measure	R^2^ (null model)	R^2^ (with group)	f^2^	Clinical difference	N (power = 90%)
GAD-7	0.090	0.114	0.027	0.8 points	392
DNA proportion	0.032	0.086	0.059	6%	181

To estimate effect size, we adopted a linear regression fixed model R^2^ increase approach, where we first computed a null model with a control predictor for the initial GAD-7 (mean-centered). We then constructed our test model that included “group” as a predictor variable, with levels for “intervention” (patients who had voluntarily installed and logged in to Limbic Care; n = 147) and “control” (patients who had never logged in to Limbic Care; n = 93). These groups are a self-selected equivalent to the randomized groups in the present study. Finally, to estimate the required sample per outcome measure, we compared the R^2^ value per model and computed the f ^2^ effect size attributable to the additional “group” predictor.

To adequately power a group × week interaction effect in our model for both our clinical and engagement measures (≥ 90% power), we selected the higher estimated sample size (N = 392 for GAD-7) which would require at least n = 196 in each arm. Given our 3:2 treatment allocation ratio, we set a slightly overestimated (+20) target n = 216 for our control arm and n = 324 for our intervention arm, giving a total target N = 540.

### Statistics and reproducibility

All analyses were conducted using Python (version 3.10.4). Linear mixed-effects models were fitted using the “statsmodels” package (version 0.14.3) and independent samples t-tests were computed using the “scipy” package (version 1.14.1). Statistical significance was set at α = 0.05 (two-tailed) except when correcting for multiple comparisons (see explanation of Bonferroni correction for primary outcomes below). Before performing any mixed effects regression analysis, residuals were assessed for normality by visual inspection. All models included random intercepts for participants.

For each mixed effects regression model, we report multiple effect size metrics. Unstandardized coefficients (b) represent the actual change in outcome measures in their original units, while standardized coefficients (β) allow for comparison across different outcome measures. We also report Cohen’s f² to measure the proportion of variance explained by specific model terms. To calculate f², we compared the variance explained (R²) by models with and without the term of interest (e.g., group × week interaction). R² was computed as the ratio of variance in model-predicted values to total outcome variance, and f² was calculated as (R²_full_ - R²_null_) / (1 - R²full). This metric allows direct comparison with our power calculations, where f² = 0.027 represented our target effect size for our clinical primary outcomes and f² = 0.059 represented our target effect size for our engagement primary outcomes (see Table 2).

We also report p-values (original and Type I error corrected, where applicable) and unstandardized 95% confidence intervals are also reported for all effects of interest. Exploratory Bayesian analyses were conducted using JASP (version 0.19.3) using default Cauchy priors (width = 0.707).

### Missing data handling

For primary outcome measures of engagement (frequency and duration), data were collected continuously and automatically and thus there were no missing data. For primary and secondary participant-reported outcomes (primary: GAD-7, PHQ-9, secondary: adverse events), missing data primarily resulted from survey non-completion (see Fig. 2 for CONSORT diagram). We handled this through maximum likelihood estimation within our linear mixed-effects models, which maintains statistical power and reduces bias under the missing at random (MAR) assumption.

For safety data, where weekly adverse event reporting was incomplete due to missed surveys, we implemented multiple imputation using chained equations (MICE) via the miceforest Python package (v6.0.3). Ten imputed datasets were created, with imputation models including all baseline characteristics and previously reported adverse events. Statistics were pooled according to Rubin’s rules, with Monte Carlo errors <10% of the respective standard errors, indicating stable imputation.

Outlier management followed a consistent protocol across measures, using a standard threshold of three standard deviations from the mean. Engagement duration outliers (≥3 SD from mean) were removed (PDF sessions: ≥148 minutes, 1.17% removed; app sessions: ≥93 minutes, 1.90% removed) as these represented instances of measurement error where participants may have left the digital workbook open on their computer or left their phone open on the app.

For exploratory analyses predicting symptom change, outliers (≥3 SD from the mean change across any of the four symptom scales were excluded from analysis (n = 9 total; 5 intervention, 4 control), as such extreme variations likely represent measurement error rather than true treatment effects. This conservative approach is consistent with established practices in clinical trials where extreme outliers can disproportionately influence treatment effect estimates and reduce statistical conclusion validity.

### Primary outcomes

For therapy engagement, we employed linear mixed-effects modelling to analyze continuous outcome measures (number of opens, duration in minutes) over time (week) and between groups (intervention or control; see Supplementary information for full model specifications). Our main coefficient of interest was the main effect of group, capturing the overall differences in engagement with the app intervention vs the control group’s digital workbook.

For analysis of symptom reduction (primary: GAD-7, PHQ-9; additional: MSQ, WSAS), we used a similar linear mixed-effects modeling approach to the above engagement analysis. In an exploratory, post-hoc analysis to complement our frequentist analyses, we conducted post-hoc Bayesian linear regressions on each participant’s change in symptom score from baseline to the final week 6 survey, separately for GAD-7 and PHQ-9. Only participants who completed the final week 6 survey were included in these Bayesian analyses (n = 414, 77.97%). Bayes factors were calculated comparing null models (baseline symptoms only) against models including group effects, providing quantification of evidence for (BF_10_) or against (BF_01_) group differences.

Across all four mixed effects regression models, all coefficient p-values were Bonferroni-corrected for four comparisons, effectively setting ɑ = 0.0125 to correct for Type I error.

### Secondary outcomes

We assessed intervention safety through systematic monitoring of adverse events, analyzing both the proportion of participants reporting any adverse events and the frequency of events per participant. Adverse events were collected through weekly surveys where participants reported any negative experiences related or unrelated to their use of the digital intervention or workbook.

We employed logistic regression to compare the proportion of participants reporting adverse events between groups, with the binary outcome of whether a participant reported any adverse event during the study period. Multiple imputation using chained equations (MICE, 10 iterations) using the “miceforest” package (version 6.0.3) addressed missing data from survey non-completion. Results were pooled according to Rubin’s rules, with reported statistics including 95% confidence intervals and p-values. We then also compared the mean number of adverse events per participant between groups using independent samples t-tests. We report results pooled across multiple imputations (MICE, 10 iterations) according to Rubin’s rule.

In an exploratory extension of the safety analysis, we conducted Bayesian equivalents of both the logistic regression and the between-group comparison of event frequencies. While not pre-registered, these analyses were added to quantify evidence for the null hypothesis of no group differences in adverse events (BF₀₁, with values ≥ 3 indicating substantial evidence for no group difference). These Bayesian analyses were conducted on complete cases only, without imputation for missing data.

### Additional outcomes

Additional clinical outcomes (MSQ and WSAS) were analyzed following the same linear mixed effects modeling approach used for the primary outcomes.

Participants’ subjective experiences with their assigned intervention were assessed through both weekly ratings (on 11 dimensions including satisfaction, perceived effectiveness, and motivation to use the materials – see Supplementary Table 17 for all items) and through the Δ change in rating from the baseline survey to the final week 6 survey (likelihood of trying human-led therapy in the future, preference for apps over digital workbooks for a wellbeing course, and trust in wellbeing apps that use artificial intelligence). The 11 weekly ratings were averaged across weeks and compared between groups with independent samples t-tests, controlling for Type I error with the False Discovery Rate (FDR) correction method. Changes in ratings over time were examined using linear regression models for three key dimensions (engagement, satisfaction, and perceived benefit). These models included fixed effects for time, group, and their interaction, with Bonferroni correction applied to p-values for the three comparisons.

### Exploratory analyses

We conducted three post-hoc, exploratory investigations examining the relationship between engagement patterns and symptom reduction. As these analyses were exploratory, p-values were not corrected for multiple comparisons and results should be interpreted as hypothesis-generating rather than confirmatory.

To investigate whether overall engagement duration moderated treatment effects, we extended our primary outcome models (GAD-7, PHQ-9) by including engagement duration as an additional predictor. Engagement was operationalized using a median split of total duration, calculated separately for each group to account for inherent differences in intervention formats. Models included three-way interactions between time, group, and engagement level (high/low), with random intercepts for participants and random slopes for time (see Supplementary Tables 13– 16 for full model specifications).

Within the intervention group only, we examined how different types of engagement related to symptom trajectories. Four distinct, continuous engagement variables were analyzed from the six-week intervention period: (i) number of messages sent in Let’s Chat, (ii) number of psychoeducation lessons completed, (iii) number of CBT exercises completed, and (iv) number of guided sessions completed. Linear mixed-effects models assessed how each engagement type modulated symptom reduction over time across all clinical measures (GAD-7, PHQ-9, WSAS, MSQ). Models included interaction terms between time and each engagement type, controlling for baseline symptoms and demographic factors that differed between engagement pattern subgroups.

In the final exploratory investigation, we identified three distinct subgroups based on observed usage patterns: (i) active control participants meeting minimum engagement criteria (≥5 minutes viewing time, ≥6 pages viewed), (ii) intervention participants who completed psychoeducation/CBT exercises without guided sessions, and (iii) intervention participants who completed guided sessions. We compared symptom trajectories across these subgroups using linear mixed-effects models, controlling for baseline characteristics that differed between subgroups (due to these not having been randomly assigned). The primary parameter of interest was the subgroup × time interaction, indicating differential rates of symptom change between engagement patterns.

## Results

### Participant recruitment and retention

We screened a total of 2146 individuals for eligibility in our study, of whom 682 participants (31.78%) met the inclusion criteria (see Participants section for inclusion/exclusion criteria and Fig. 2). Of these, 540 participants participated in the baseline survey where they were randomly assigned to one of two groups with a 3:2 ratio: 322 participants to the intervention group and 218 participants to the control group. In a post-hoc decision to preserve the scientific integrity of our group comparisons, we excluded 9 participants (4.1%) from further analysis who had been exposed to both interventions. This modification to our analysis sample resulted in a modified Intention to Treat (mITT) population of 209 participants in the active control group, and all 322 participants in the intervention group.

Baseline characteristics of the study participants are presented in Supplementary Data 1. The active control group (n = 209) and intervention group (n = 322) showed similar demographic compositions. The majority of participants in both groups were female (72.3% in control, 69.6% in intervention) and white (70.3% in control, 69.6% in intervention). Mean age was 36.0 years (SD = 11.5) in the control group and 37.6 years (SD = 11.8) in the intervention group. Most participants were employed full-time (42.1% control, 51.6% intervention) and were not students (72.3% control, 71.7% intervention). Regarding clinical characteristics, mean GAD-7 scores were 10.13 (SD = 4.25) in the control group and 9.15 (SD = 4.32) in the intervention group. Mean PHQ-9 scores were 10.67 (SD = 5.53) and 9.87 (SD = 4.87) for control and intervention groups respectively. Prior therapy experience was reported by 52.7% of control group participants and 53.0% of intervention group participants, while 21.5% of control and 22.7% of intervention group participants reported current use of any psychoactive medication.

Throughout the six-week study period, participants from both groups were invited to complete a weekly survey to monitor their progress and engagement while they had access to their allocated materials (the Limbic app or a digital workbook). Participants in both arms were encouraged to engage with the CBT materials four times per week throughout the study. Importantly, however, they were not compensated for their engagement with these materials and it was clearly communicated that engagement was not mandatory or related to their compensation – participants were only compensated for completing the weekly surveys. The average weekly survey completion rates were high and comparable between the groups, indicating strong participant retention: 85.40% ± 4.76% for the intervention group (n = 322) and 84.87% ± 4.60% for the control group (n = 209). By the final week (Week 6), retention rates remained consistent, with 82.92% of the intervention group and 83.01% of the control group completing the survey.

### Enhanced therapy engagement for app than digital workbook

#### Engagement frequency

**Fig. 3:**
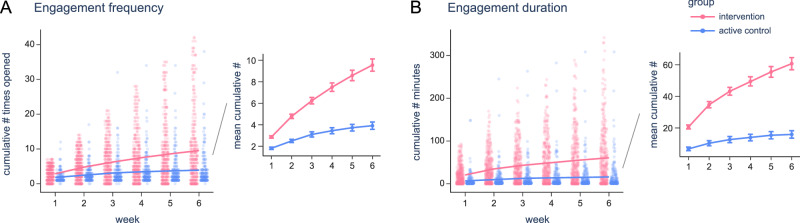
Increased engagement with the Limbic App. **A** Cumulative number of opens automatically logged by the app (intervention: pink) or digital workbook (control: blue) per week. Dots represent individual participants (n = 522), with the line representing each group mean. The right-hand graph displays a zoomed in view of the mean per group, with error bars indicating standard error. **B** Same as (**A**), except for the cumulative engagement duration (in minutes) instead of number of opens (n = 522).

First, we compared how frequently participants engaged with the therapeutic materials by examining the number of times the digital workbook was opened in the control group versus the app in the intervention group (see Fig. 3A). These metrics were objectively measured through passive digital tracking systems built into both the app and the digital workbook platform. In the control group (n = 209), 21 participants (10.05%) reported printing the digital workbook out at some point during the study. As we could not track engagement with printed copies, the following engagement frequency metrics may underestimate total engagement for the group. On average, control participants opened the digital workbooks 3.9 ± 5.0 times over the six-week period, with the number of opens per week declining over time — from 1.8 ± 1.5 times in Week 1 down to 0.2 ± 0.7 times in Week 6. In contrast, intervention participants (n = 322) opened the app a total of 9.6 ± 10.2 times over six weeks, more than double (2.4 times) the frequency of the control group (main effect of group: b = -1.199, β = -0.318, *p* <.001, p_bonf_ < .001, 95% CI = [-1.488, -0.910], f^2^ = .073; see Supplementary Table 1 for model output). Similar to the control group, the number of app opens per week decreased over time, from 2.9 ± 2.0 times in Week 1 (1.6 times higher than the control group) to 1.0 ± 1.8 times in Week 6 (5 times higher than the control group). By the final week of the study, the app was opened 5 times more frequently than the digital workbook.

#### Engagement duration

We assessed the total duration participants spent engaging with the therapeutic materials (see Fig. 3B), as measured through automatic tracking. The active control group (n = 209) viewed the digital workbook on their devices for a total of 15.9 ± 34.0 minutes over the six weeks. The longest viewing time occurred in Week 1 (6.8 ± 16.4 minutes), declining sharply to just 0.5 ± 2.5 minutes by Week 6. In contrast, intervention participants (n = 322) spent a total of 60.7 ± 69.0 minutes using the app, 3.8 times longer than the control group (main effect of group: b = -13.977, β = -0.455, *p* < 0.001, p_bonf_ < 0.001, 95% CI = [-16.307, -11.646], f^2^ = .067; see Supplementary Table 2 for model output). App engagement was highest in Week 1 (20.6 ± 22.1 minutes, 3 times higher than control) and decreased over time to 5.2 ± 12.0 minutes in Week 6 (10.4 times higher than control).

### Similar symptom reduction across groups

**Fig. 4:**
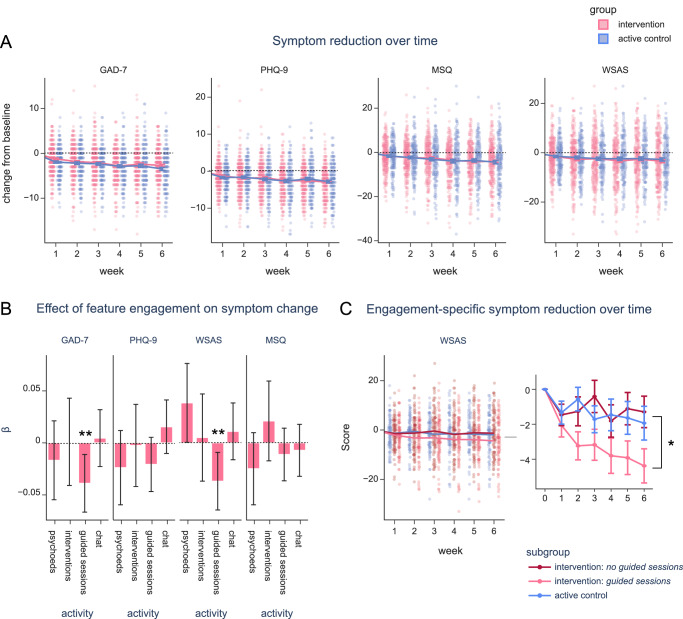
Symptom reduction over time. **A** Generalized Anxiety Disorder score (GAD-7), Patient Health Questionnaire (PHQ-9) score, Work and Social Adjustment Scale (WSAS) score, and Mini Sleep Questionnaire (MSQ) scores each week (x axis), with dots representing individuals from each group (intervention: pink, control: blue, n = 522). Lines and markers indicate the mean per group, with error bars indicating standard error.** B** Beta (β) coefficients for the effect of feature engagement (number of psychoeducation lessons or “psychoeds”, CBT activities or “interventions”, guided sessions, or messages sent in “let’s chat”) on the change in symptom scores over time, taken from separate linear mixed-effects models per measure (GAD-7, PHQ-9, WSAS, and MSQ; n = 284). Error bars indicate the 95% confidence interval of the standardized model coefficients. ** p < 0.01, • p < 0.10 **C** WSAS scores for participants in the control group (blue) with sufficient activity (at least 5 minutes of viewing, and viewed at least 6 pages), participants in the intervention group who did guided sessions (pink), and participants in the intervention group who did not do guided sessions but did at least one psychoeducation lesson or CBT activity (pink, dashed). Dots represent individual participants (n, lines and markers indicate the mean per subgroup, and error bars indicate the standard error. The pop-out graph displays a zoomed-in view of the subgroup means. * p < 0.05, from linear mixed effects model predicting symptom score (x axis, as separate models) by a group (control, guided sessions, other activities) × time (week) interaction.

Our other primary outcome measures focused on symptom reduction, specifically anxiety symptoms measured by the GAD-7 and depression symptoms measured by the PHQ-9. Both groups exhibited a reduction in anxiety and depression symptoms over the six-week period, suggesting that the intervention was comparable to the active control in terms of clinical efficacy (main effect of week on GAD-7: b = -0.432, β = -0.183, *p* < 0.001, 95% CI = [-0.491, -0.374], f^2^ < .001; main effect on PHQ-9: b = -0.442, β = -0.159, *p* < 0.001, 95% CI = [-0.505, -0.378], f^2^ < .001; see Fig. 4A and Supplementary Tables 3, 4 for model output). The intervention group (n = 317) showed a mean reduction (from baseline to the most recent data collected per participant) of -2.46 ± 3.94 on the GAD-7 (d = 0.62) and -2.67 ± 4.09 on the PHQ-9 (d = 0.65). Similarly, the control group (n = 205) demonstrated a mean reduction of -3.13 ± 4.09 on the GAD-7 (d = 0.76) and -2.68 ± 4.55 (d = 0.59) on the PHQ-9. These rates of decline were comparable between groups for GAD-7 (group × week: b = -0.032, β = -0.003, p = 0.505, p_bonf_ = > 0.999, 95% CI = [-0.125, 0.061]) and PHQ-9 (group × week: b = 0.052, β = 0.004, p = 0.315, p_bonf_ = > 0.999, 95% CI = [-0.049, 0.153]). In a post-hoc Bayesian regression analysis to quantify the evidence for the null hypothesis, we found moderate evidence that both groups were equivalent on both anxiety (BF_01_ = 5.985) and depression (BF_01_ = 5.861) reduction by the time of the final week 6 survey (intervention: n = 248, active control: n = 166).

We also assessed other clinically relevant measures: the Work and Social Adjustment Scale (WSAS)^24^ for general well-being and the Mini Sleep Questionnaire (MSQ)^25^ for sleep disorder symptoms. Like the GAD-7 and PHQ-9, both of these measures reduced significantly throughout the study period across participants in both groups (main effect of week on WSAS: b = -0.475, β = -0.098, *p* < 0.001, 95% CI = [-0.595, -0.355], f^2^ < .001; main effect of week on MSQ: b = -0.650, β = -0.123, *p* < 0.001, 95% CI = [-0.158, 0.224], f^2^ < .001), and there were no significant differences between the intervention and control groups (main effect of group on WSAS: b = -0.145, β = -0.007, p = 0.789, 95% CI = [-1.209, 0.919]; group × week effect on WSAS: b = 0.057, β = 0.003, p = 0.556, 95% CI = [-0.134, 0.249]; main effect of group on MSQ: b=-0.428, β = -0.019, p = 0.439, 95% CI = [-1.510, 0.654]; group × week effect on MSQ: b = 0.033, β = 0.002, p = 0.734, 95% CI = [-0.158, 0.224]; see Supplementary Tables 5, 6). To follow-up these non-significant differences between groups, we again conducted a post-hoc Bayesian linear regression and found moderate to strong evidence for the null hypothesis that both groups showed equivalent reduction in WSAS (BF_01_ = 5.707) and MSQ (BF_01_ = 15.252) from baseline to the final week 6 survey (intervention: n = 248, active control: n = 166).

### Similar safety between intervention and control groups

Ensuring the safety of participants is crucial when introducing new therapeutic interventions, especially those involving emerging technologies like genAI. Throughout the study period, we closely monitored the occurrence of self-reported adverse health events, which participants were asked to report regardless of whether they were related to the intervention or not, including both physical or mental health. All reports were monitored on a weekly basis by clinical researchers so that any severe events would be detected and the participant could be referred to crisis support (this was never required).

Reported events fell into three main categories: physical health (e.g., injuries, infections, chronic conditions), mental health (e.g., anxiety, depression), and life stressors (e.g., work stress, family conflicts). Of the 190 events reported in the intervention group, only one (0.05%) mentioned the intervention negatively (work stress not relieved by the app). In contrast, several reports mentioned the intervention helping with difficulties (e.g., “The app did help” during panicky feelings). Importantly, no events met ISO 14155:2020 criteria for a Serious Adverse Event (SAE).

The proportion of participants reporting any adverse event was comparable between groups: 36.96% (intervention, n = 322) versus 38.28% (control, n = 209; b = 0.018, β = 0.055, p = 0.885, 95% CI = [0.797,1.301]), with substantial evidence for no group difference (BF_01_ = 8.878). Similarly, the average number of events per participant showed no significant difference between intervention (0.62 events) and control groups (0.72 events; t = -1.239, *p* = 0.221, BF_01_ = 4.242). Thus, the genAI-enabled app had a comparable safety profile to digital workbooks.

### Higher usability and satisfaction for the genAI intervention

Throughout the study period, we surveyed participants in both groups weekly to gather their impressions of the tool to which they had been assigned (see Supplementary Table 17 for a list of questionnaire items). We focused on key aspects such as usability, satisfaction, and perceived learning to comprehensively evaluate the user experience.

On average across all six weeks, participants allocated to the Limbic app (n = 322) reported significantly higher scores on accessibility (t = 4.194, *p* < 0.001, p_FDR_ < .001, d = 0.16), ease of use (t = 10.245, *p* < 0.001, p_FDR_ < .001, d = 0.40), motivation to engage with the intervention (t = 2.832, *p* = 0.005, p_FDR_ = .013, d = 0.11), and how personalized the intervention felt (t = 14.765, *p* < 0.001, p_FDR_ < .001, d = 0.56). Other aspects, such as a sense of satisfaction and the perceived usefulness of the intervention, were not significantly different between groups (see Supplementary Table 18 and Supplementary Fig. 1A–C).

We also examined how key subjective measures changed from baseline to the final week of the study across the two groups. Only participants in the intervention group (n = 267 who completed the final survey) reported an increase in their trust in AI-powered wellbeing apps over the six-week period (group × week: b = -0.408, β = -0.208, 95% CI = [-0.590, 0.226], *p* < 0.001, p_bonf_ < .001; Supplementary Table 19). The overall preference for using an app over a digital workbook increased for both the intervention group and active control group (n = 174 who completed the final survey; main effect of week: b = 0.487, β = 0.157, 95% CI = [0.260, 0.715], *p* < 0.001, p_bonf_ < .001), with no difference between groups (group × week: b = -0.117, β = -0.037, 95% CI = [-0.479, 0.245], *p* = 0.527, p_bonf_ > .999). Similarly, participants in both groups felt they were more likely to try human-led therapy after the trial was over (main effect of week: b = 0.536, β = 0.286, 95% CI = [0.410, 0.661], p <.001, p_bonf_ < .001), with no significant difference between groups (group × week: b = -0.041, β = -0.022, 95% CI = [-0.241, 0.159], *p* = 0.686, p_bonf_ > .999; see Supplementary Fig. 1D).

### Exploratory analyses

#### Positive user feedback on AI helpfulness and safety

To gain a better understanding of intervention-specific effects on safety, we examined participant feedback provided for individual conversations with the AI. After each conversation within the app, intervention participants could rate messages as “helpful,” “unhelpful,” or “harmful.” Of the 1222 conversations that received ratings, 615 (50.33%) were rated as helpful, 105 (8.59%) as unhelpful, and only 1 (0.08%) as harmful. The single conversation rated as harmful was reviewed by a board-certified cognitive-behavioral therapist who determined the AI behavior was benign. These results provide additional support for the safety profile of the AI-enabled intervention, complementing our primary adverse event analyses.

#### Higher engagement relates to greater symptom reduction

Having observed similar symptom reduction between the intervention and active control groups, we conducted an exploratory investigation into whether participants who engaged more with either the app or digital workbook also saw greater benefits to their mental health. While we acknowledge that any relationships observed would be correlational rather than causal, we reasoned that if there is no relationship between engagement and symptom reduction, then this might suggest that we had merely observed a regression to the mean in both groups. On the other hand, an improvement in symptoms in those who engaged more with either the digital workbook or the app is suggestive of meaningful clinical impact.

To test this, we modelled symptom reduction over time per group, moderated by engagement duration (median split of total duration per participant). We discovered that anxiety and depression decreased at a faster rate for participants who engaged with their materials for longer (engagement × week: GAD-7 model – b = 0.153, β = 0.032, 95% CI = [0.037, 0.269], *p* = 0.010; PHQ-9 model – b = 0.142, β = 0.026, 95% CI = [0.015, 0.268], *p* = 0.028), regardless of whether they were in the intervention or control group (group × engagement × week: GAD-7 model – b = -0.124, β = -0.013, 95% CI = [-0.309, 0.061], *p* = 0.190; PHQ-9 model – b = -0.118, β = -0.010, 95% CI = [-0.319, 0.083], *p* = 0.250; see Supplementary Fig. 2 and Supplementary Tables 7, 8). Therefore, these exploratory results suggest that the reduction in GAD-7 and PHQ-9 we observed in both groups were likely related to engagement with the therapeutic materials rather than being solely attributable to non-specific effects like regression to the mean.

#### Quality of app engagement and symptom reduction

Recognizing that overall group comparisons might mask the effects of individual engagement with specific app features, we conducted an exploratory investigation into whether feature engagement predicted symptom reduction within the intervention group. The activities a user could do in the app included: 1) reading psychoeducation material and clarifying their understanding of the content by engaging with the conversational agent, 2) working through an interactive CBT exercise with the conversational agent, 3) engaging in open-ended conversation with the “Let’s Chat” feature, and 4) working through a “guided session” with the conversational agent, which allows users to explore specific issues or challenges they are experiencing through guided questioning that leads them to a CBT exercise tailored to their needs.

We investigated whether engaging with each of these four features (i.e., starting a psychoeducation lesson, CBT activity, guided session, or a conversation) could predict improvements in anxiety (GAD-7), depression (PHQ-9), sleep problems (MSQ), and overall well-being (WSAS). We separately modeled changes in these outcome measures over time as a function of the number of times a participant had engaged with each feature (see Supplementary Tables 9-12 for model specification and output).

These models revealed that intervention participants who engaged more with the guided sessions showed significantly greater improvement in anxiety over time than those who engaged less (guided sessions × week interaction on GAD-7: b = -0.092, β = -0.039, p = 0.006, 95% CI = [-0.157, -0.026]; see Fig. 4B). WSAS scores, reflecting general impairments in well-being, also improved most for participants who completed guided sessions (guided sessions × week: b = -0.191, β = -0.038, p = 0.007, 95% CI = [-0.220, -0.051]). This effect was not observed for depression symptoms (PHQ-9, *p* > 0.05). Notably, the enhanced improvements in anxiety and well-being were specific to the guided sessions, with no other feature having significant modulatory effects (all p > 0.052).

In a final, follow-up exploratory step, we compared symptom reduction profiles between intervention participants who engaged with guided sessions (“guided sessions” subgroup: n = 94) to active control participants who actively engaged with the digital workbooks (“active control” subgroup: n = 90). We also included a subgroup of intervention participants who engaged with other app features (“no guided sessions” subgroup: n = 94) in the comparison (see Supplementary Results for subgroup specification and baseline characteristics). We discovered that, while symptoms of anxiety, depression, and sleep problems reduced at a similar rate for all participants regardless of their engagement pattern (p > 0.299; for all subgroups × week interactions; see Supplementary Tables 13–16 for model output, and Fig. 4C), participants who voluntarily engaged with guided sessions showed significantly greater improvements in overall well-being than those who engaged with the digital workbooks (guided sessions subgroup × week: b = -0.365, β = -0.060, *p* = 0.014, 95% CI = [-0.656, -0.073]). Importantly, this effect was significant even after accounting for the total duration of app or workbook engagement per participant. This post-hoc analysis tentatively suggests that, while engagement with this specific genAI feature did not differentially impact anxiety or depression symptoms beyond basic digital materials, it may confer an additional benefit to a user’s overall sense of wellbeing.

## Discussion

In this RCT, we compared a genAI-enabled therapy delivery tool (intervention) to digital workbooks delivering static CBT content (active control), a common delivery format of self-directed care in mental health therapy. The intervention significantly increased participant engagement while maintaining comparable safety standards to the active control condition. We did not observe statistically significant overall differences in anxiety and depression symptom reduction between groups. However, exploratory analyses suggested that participants who self-selected to engage with the app’s AI-driven clinical personalization feature experienced stronger anxiety (but not depression) symptom reduction (within the intervention group) and improved overall wellbeing compared to the active control group. Overall, these findings suggest that the controlled implementation of genAI can positively and safely enhance participant engagement, but that engagement with a stand-alone genAI tool may not directly translate into clinical symptom improvement. Additional strategies, such as encouraging broader uptake of clinically-personalized features or human supervision^26^, might be required to achieve clinical improvement with genAI-enabled therapy delivery tools.

A significant hurdle in CBT is encouraging patients to consistently engage with therapeutic homework, which is thought to be essential for the treatment’s effectiveness^9–11^. Our study tackles this pervasive problem by demonstrating that delivering CBT activities through an interactive, genAI-enabled app increases participant engagement compared to digital workbooks. The app was opened 2.4 times more frequently and overall engagement duration was 3.8 times longer. The app also received small but statistically significant boosts in usability (Cohen’s d = 0.40) and personalization (Cohen’s d = 0.56) ratings compared to the active control condition, two components that are thought to be crucial for sustaining engagement with mental health apps^10^. This aligns with and expands upon previous research showing positive attitudes towards chatbot-delivered CBT^26^ and increased engagement with chatbots compared to static materials^27^.

These engagement benefits magnified over the study period, suggesting that engagement with genAI is especially advantageous for usage over longer periods of times (weeks or months), aligning with the waiting periods typically endured by patients on waitlists for psychotherapy. Crucially, our experimental design was intentionally devoid of external guidance regarding app interaction, reminders, or external encouragement (with the only indirect reminder being the weekly surveys to collect outcome measures). This approach was chosen to maximize the experimental validity of the relative comparison of engagement between the study arms.

Nevertheless, the absolute engagement metrics we observed for the interventions matched or exceeded those reported in previous studies on chatbots designed to improve wellbeing in non-clinical samples – for example, a study reporting that users opened a smoking cessation chatbot app once per week on average^28^, and another reporting that users engaged with a chatbot for depression for 25 minutes on average over six weeks^29^. Direct cross-study comparisons are, however, inherently challenging due to significant heterogeneity in app functionalities, target populations, and engagement reporting^30^. Engagement metrics are typically higher in studies involving clinical populations with inherently greater motivation^30^, significant clinician oversight and reminders^31–33^, or explicit financial incentives for app use^34^. By recruiting from a non-clinical population and deliberately omitting these external drivers of engagement, our study design enabled a scientifically rigorous comparison between our intervention and active control conditions, allowing us to more effectively isolate and attribute differences in engagement to genAI-enabled delivery of therapeutic curriculum. Thus, this establishes a conservative yet realistic baseline for autonomous user engagement in unsupervised settings (pertinent to therapy waitlists), and evidences genAI’s ability to enhance engagement with therapeutic materials compared to simpler digital formats.

Although users engaged more with the Limbic app, there were no significant group differences in symptom reduction for anxiety (GAD-7), depression (PHQ-9), sleep disturbances (MSQ), or general well-being (WSAS). Such non-significant differences in outcomes are frequently observed in RCTs that use active control conditions^35^ with effect sizes on par with the ones observed here^36^, and this pattern of results aligns with those reported by similar studies comparing digital solutions to static CBT material^37,38^. However, we had hypothesized that the enhanced engagement fostered by the clinical genAI might translate into superior symptom improvement. One possibility is that the observed improvements in both groups reflect non-specific effects such as regression to the mean, natural recovery, or placebo effects, particularly given the absence of an inactive (e.g., waitlist or sham) control. However, two pieces of evidence suggest an active therapeutic component was at play. Firstly, the observed reductions seen across groups (2.7 points on GAD-7 and PHQ-9) exceeded typical reductions seen in waitlist control conditions^31,32^.

A second, more compelling explanation may lie in the intentionally unguided nature of our experimental design which, as explained above, provided an unbiased measure of autonomous engagement but meant that participants did not receive specific instructions on how to optimally utilize the app’s features for symptom relief. This interpretation is tentatively supported by our exploratory analysis of engagement patterns, which revealed that participants engaging with the “guided sessions” feature – a genAI-heavy feature designed to closely replicate a patient-therapist interaction – experienced significantly greater improvements in well-being. This exploratory finding generates hypotheses about the potential of specific genAI interactions, when utilized. Relatedly, in a separate observational study of the same app (Limbic Care) we found pronounced clinical benefits when the app was offered to patients undergoing human-led group therapy, with weekly guidance from clinicians directing patients towards relevant app content^26^. Although these findings were observational and not causal, they align with other studies showing that personalized CBT interventions improve therapy adherence^33,39^ and that engagement with LLM-delivered CBT content under human supervision can improve mental health^35,40^. Altogether, this evidence base suggests that targeted guidance towards clinical potent features, especially within supervised clinical pathways, might be critical for translating genAI’s engagement advantages into demonstrable clinical symptom reduction – an avenue for subsequent confirmatory trials.

This RCT demonstrated that a genAI-enabled app has a comparable safety profile to digital workbooks, a format often used as self-directed treatment or to deliver homework between sessions in care settings. This finding is critical, directly addressing concerns about unpredictable or harmful responses from AI in the sensitive domain of mental health, where generic LLM safety guardrails are insufficient. Limbic Care’s safety was achieved through clinical AI safety layers to screen user input and LLM output, detecting any safety-relevant situations and handling them accordingly^20^. Moreover, to ensure consistent therapeutic quality over time, the system uses fixed, versioned LLM deployments that maintains reliable performance regardless of potential changes in underlying LLM capabilities. This study therefore suggests that generative AI can be utilized safely, as benchmarked against static materials.

A key benefit emerging from this research is the utility of genAI-enabled CBT applications as standalone tools for enhancing user engagement, particularly in unsupervised contexts such as during waitlists for psychotherapy. In these scenarios, where direct clinical oversight may be limited or delayed, the observed ability of genAI to sustain user interaction is intrinsically valuable. Such engagement might, for instance, keep patients engaged with therapy and the healthcare service, thereby potentially reducing drop-out rates^26^. Moreover, ongoing dialogue with the genAI app may enable ongoing, automatic crisis detection (e.g., through open-ended dialogue with the chatbot) to allow for more continuous monitoring of patient risk. The demonstrated safety profile of the Limbic Care app, comparable to static digital workbooks, underscores the feasibility of implementing these tools in resource-constrained healthcare systems facing staff shortages or extensive waiting periods, without requiring intensive clinical supervision.

While genAI shows considerable promise for autonomous engagement, maximizing significant clinical symptom reduction may necessitate a more symbiotic relationship between the patient, AI, and a human therapist. The optimal role for genAI in achieving substantial clinical change might be less as a therapist replacement and more as a clinical amplifier or adjunct tool. This is a potential pathway in which genAI tools could serve to enhance, rather than supplant, the human element in mental healthcare. Investigating the efficacy of such blended care models will be a fruitful avenue for future research.

A limitation of our study design is that the digital workbook used as the active control was developed specifically for this trial by members of the same organization that created the genAI intervention, which could have introduced bias in the construction of the control condition, and was not independently validated in prior standalone efficacy studies. This approach was chosen deliberately to ensure direct content parity between the study arms and thereby strengthen the internal validity of our causal claims. To ensure the comparator was robust and clinically relevant, its structure and content were closely modeled on evidence-based materials used in the UK’s NHS Talking Therapies program and developed by certified CBT therapists. Nonetheless, to minimize any potential biases, future studies comparing genAI-enabled CBT to standard care should employ independent validation or select routinely implemented approaches (e.g., therapist-delivered homework, established iCBT programs) to further clarify the generalizability of the findings seen here.

Random allocation of participants to the intervention and an active control allows us to draw causal conclusions about the impact of genAI-enabled therapy delivery on engagement and treatment outcomes. One limitation, however, is that our evidence for increased well-being among those who engaged with guided sessions was observational rather than experimentally manipulated, as a function of it being an exploratory analysis. Unmeasured factors, such as participants’ inherent motivation levels, could have contributed to the observed improvements in clinical outcomes. Future research can methodically investigate the impact of these specific clinical features on engagement and clinical outcomes by randomly assigning participants to have, or not have, access to different features.

Our study sample consisted of adults with elevated levels of self-reported anxiety and/or depression symptoms, as measured by widely-used screening tools (GAD-7 and PHQ-9) rather than clinical diagnostic assessments. Similarly, the participants in our study were not actively seeking mental healthcare (i.e., they were not patients in a healthcare system). While these screening measures used in our study are standard in many healthcare settings, the absence of clinician-administered diagnostic interviews means that our participants may not be directly comparable to clinically diagnosed populations. Additionally, the absence of a waitlist control group means we cannot definitively quantify how much of the observed symptom improvement in either group was due to natural recovery or regression to the mean rather than intervention effects.

## Conclusion

In summary, this RCT has demonstrated that genAI-enabled therapy support has a significant and beneficial effect on increasing engagement with therapeutic exercises and materials. Moreover, use of this tool was safe when benchmarked against an active control designed to approximate self-directed CBT workbooks often used within or alongside standard care. We found that a genAI-enabled therapy support as a stand-alone tool did not improve clinical outcomes compared to digital workbooks over six weeks of self-directed usage. However, exploratory analyses suggested that engagement with features providing clinical personalization, made possible with genAI, was associated with stronger anxiety symptom reduction (within the intervention group) and enhanced overall wellbeing, providing a promising avenue for future research. These results suggest that clinical AI is an effective solution for improving engagement with CBT.

## Supplementary information

Supplementary Information

Description of Additional Supplementary files

Supplementary Data 1

Supplementary Data 2

Supplementary Data 3

## Data Availability

All data generated or analyzed during this study are included in Supplementary Data 3.
