# Supplementary material for: Increasing engagement with cognitive-behavioral therapy (CBT) using generative AI: a randomized controlled trial (RCT)
Source: Commun Med (Lond). 2026 Jan 15;6:129. doi: 10.1038/s43856-025-01321-8 (PMC12953620; doi:10.1038/s43856-025-01321-8)
Supplement: Supplementary file 1 — Supplementary Information [file 43856_2025_1321_MOESM1_ESM.pdf]

Supplementary information

Model output tables – full sample

Model formula: number of opens ~ group × week + (1|subject)

Parameter	b	β	SE	95% CI	p	p_{bonf}
(intercept)	2.811	-	0.092	[2.630, 2.992]	6.914-203***	2.765e-202***
group	-1.199	-0.318	0.147	[-1.488, -0.910]	4.193e-16***	1.677e-15***
week	-0.348	-0.323	0.014	[-0.375, -0.322]	7.043-146***	2.817e-145***
group × week	0.074	0.020	0.022	[0.032, 0.117]	5.887e-04***	0.002**

Supplementary Table 1. Linear mixed effects model of usage frequency (number of opens) over time (weeks) as a function of group (intervention or control). β = coefficient, SE = standard error, CI = confidence interval, * $p < 0.05$, ** $p < 0.01$, *** $p < .001$, p_{bonf} = Bonferroni correction for 4 comparisons across primary outcomes

Model formula: usage duration (minutes) ~ group × week + (1|subject)

Parameter	b	β	SE	95% CI	p	p_{bonf}
(intercept)	20.480	-	0.746	[19.018, 21.942]	6.021e-166***	2.409e-165***
group	-13.977	-0.455	1.189	[-16.307, -11.646]	6.640e-32***	2.656e-31***
week	-2.962	-0.337	0.148	[-3.251, -2.672]	3.002-89***	1.201e-88***
group × week	1.860	0.061	0.236	[1.398, 2.322]	2.931-15***	1.172e-14***

Supplementary Table 2. Linear mixed effects model of usage duration (minutes) over time (weeks) as a function of group (intervention or control). b = unstandardized coefficient, β = standardized coefficient, SE = standard error, CI = confidence interval, * $p < 0.05$, ** $p < 0.01$, *** $p < .001$, p_{bonf} = Bonferroni correction for 4 comparisons across primary outcomes

Model formula: GAD-7 ~ group × week + baseline GAD-7 + (1|subject)

Parameter	b	β	SE	95% CI	p	p_{bonf}
(intercept)	8.863	-	0.159	[8.552, 9.174]	0.000e+00***	0.000e+00***
group	-0.035	-0.004	0.254	[-0.532, 0.462]	0.890	1
week	-0.432	-0.183	0.030	[-0.491, -0.374]	1.629e-47***	6.516e-47***
group × week	-0.032	-0.003	0.048	[-0.125, 0.061]	0.505	1
baseline GAD-7	0.763	0.689	0.025	[0.715, 0.812]	4.642e-207***	1.857e-206***

Supplementary Table 3. Linear mixed effects model of GAD-7 (Generalized Anxiety Disorder) over time (weeks) as a function of group (intervention or control). b = unstandardized coefficient, β = standardized coefficient, SE = standard error, CI = confidence interval, * $p < 0.05$, ** $p < 0.01$, *** $p < .001$, p_{bonf} = Bonferroni correction for 4 comparisons across primary outcomes

Model formula: PHQ-9 ~ group × week + baseline PHQ-9 + (1|subject)

Parameter	b	β	SE	95% CI	p	p_{bonf}
(intercept)	9.569	-	0.176	[9.225, 9.914]	0.000e+00***	0.000e+00***
group	-0.044	-0.004	0.281	[-0.594, 0.507]	0.876	1
week	-0.442	-0.159	0.032	[-0.505, -0.378]	1.495e-42***	5.981e-42***
group × week	0.052	0.004	0.051	[-0.049, 0.153]	0.315	1
baseline PHQ-9	0.801	0.737	0.023	[0.756, 0.847]	1.659e-260***	6.635e-260***

Supplementary Table 4. Linear mixed effects model of PHQ-9 (Patient Health Questionnaire) over time (weeks) as a function of group (intervention or control). b = unstandardized coefficient, β = standardized coefficient, SE = standard error, CI = confidence interval, * $p < 0.05$, ** $p < 0.01$, *** $p < .001$, p_{bonf} = Bonferroni correction for 4 comparisons across primary outcomes

Model formula: WSAS ~ group × week + baseline WSAS + (1|subject)

Parameter	b	β	SE	95% CI	p
(intercept)	16.436	-	0.349	[15.751, 17.120]	0.000e+00***
group	0.203	0.010	0.557	[-0.889, 1.295]	0.716
week	-0.477	-0.098	0.061	[-0.597, -0.356]	7.215e-15***
group × week	0.058	0.003	0.098	[-0.133, 0.250]	0.549
baseline WSAS	0.786	0.674	0.029	[0.730, 0.842]	1.797e-164***

Supplementary Table 5. Linear mixed effects model of WSAS (Work and Social Adjustment Scale) over time (weeks) as a function of group (intervention or control). b = unstandardized coefficient, β = standardized coefficient, SE = standard error, CI = confidence interval, * $p < 0.05$, ** $p < 0.01$, *** $p < .001$

Model formula: MSQ ~ group × week + baseline MSQ + (1|subject)

Parameter	b	β	SE	95% CI	p
(intercept)	22.641	-	0.347	[21.960, 23.321]	0.000e+00***
group	-0.212	-0.010	0.554	[-1.297, 0.874]	0.702
week	-0.652	-0.123	0.061	[-0.773, -0.532]	1.699e-26***
group × week	0.035	0.002	0.098	[-0.157, 0.226]	0.723
baseline MSQ	0.818	0.725	0.025	[0.769, 0.867]	5.751e-234***

Supplementary Table 6. Linear mixed effects model of MSQ (Mini Sleep Questionnaire) over time (weeks) as a function of group (intervention or control). b = unstandardized coefficient, β = standardized coefficient, SE = standard error, CI = confidence interval, * $p < 0.05$, ** $p < 0.01$, *** $p < .001$

Model formula: GAD-7 ~ group × week × engagement + baseline GAD-7 + (1|subject)

Parameter	b	β	SE	95% CI	p
(intercept)	8.759	-	0.222	[8.324, 9.194]	0.000e+00***
group	0.151	0.015	0.356	[-0.547, 0.849]	0.671
engagement (low)	0.159	0.017	0.317	[-0.462, 0.781]	0.615
group × engagement	-0.347	-0.035	0.506	[-1.338, 0.644]	0.492
week	-0.494	-0.209	0.040	[-0.572, -0.416]	1.440e-35***
group × week	0.014	0.001	0.064	[-0.112, 0.139]	0.829
engagement × week	0.153	0.032	0.059	[0.037, 0.269]	0.010**
group × engagement × week	-0.124	-0.013	0.094	[-0.309, 0.061]	0.190
baseline GAD-7	0.765	0.690	0.025	[0.716, 0.814]	1.685e-206***

Supplementary Table 7. Linear mixed effects model of GAD-7 over time (weeks) as a function of group (intervention or control) and engagement duration (median split of total minutes per participant). b = unstandardized coefficient, β = standardized coefficient, SE = standard error, CI = confidence interval, * $p < 0.05$, ** $p < 0.01$, *** $p < .001$

Model formula: PHQ-9 ~ group × week × engagement + baseline GAD-7 + (1|subject)

Parameter	b	β	SE	95% CI	p
(intercept)	9.438	-	0.247	[8.954, 9.922]	0.000e+00***
group	0.188	0.016	0.395	[-0.585, 0.962]	0.633
engagement (low)	0.254	0.023	0.352	[-0.436, 0.944]	0.471
group × engagement	-0.502	-0.044	0.561	[-1.601, 0.597]	0.371
week	-0.512	-0.185	0.043	[-0.597, -0.427]	3.427e-32***
group × week	0.117	0.010	0.070	[-0.020, 0.254]	0.093
engagement × week	0.142	0.026	0.065	[0.015, 0.268]	0.028*
group × engagement × week	-0.118	-0.010	0.103	[-0.319, 0.083]	0.250
baseline PHQ-9	0.802	0.738	0.023	[0.757, 0.848]	4.562e-260***

Supplementary Table 8. Linear mixed effects model of PHQ-9 over time (weeks) as a function of group (intervention or control) and engagement duration (median split of total minutes per participant). b = unstandardized coefficient, β = standardized coefficient, SE = standard error, CI = confidence interval, * $p < 0.05$, ** $p < 0.01$, *** $p < .001$

Model formula: GAD-7 ~ psychoeducation × week + CBT exercises × week + guided sessions × week + conversations × week + baseline GAD-7 + (1|subject)

**Parameter**	**b**	**β**	**SE**	**95% CI**	**p**
(intercept)	7.298	-	0.136	[7.031, 7.565]	0.000e+00***
conversations	0.002	0.006	0.014	[-0.024, 0.029]	0.867
week	-0.429	-0.184	0.029	[-0.486, -0.372]	1.058e-48***
conversations × week	9.329E-04	0.005	0.003	[-0.005, 0.007]	0.743
CBT exercises	-0.065	-0.033	0.099	[-0.259, 0.129]	0.514
CBT exercises × week	9.113E-04	9.431E-04	0.021	[-0.040, 0.041]	0.965
psychoeducation	-0.054	-0.033	0.076	[-0.204, 0.095]	0.476
psychoeducation × week	-0.014	-0.017	0.016	[-0.045, 0.018]	0.386
guided sessions	-0.054	-0.011	0.160	[-0.367, 0.260]	0.738
guided sessions × week	-0.092	-0.039	0.033	[-0.157, -0.026]	0.006**
baseline GAD-7	0.745	0.685	0.032	[0.683, 0.807]	2.235e-122***

Supplementary Table 9. Linear mixed effects model of GAD-7 (Generalized Anxiety Disorder) over time (weeks) as a function of app feature engagement (psychoeducation, CBT exercises, guided sessions, or "Let's chat" conversations). b = unstandardized coefficient, β = standardized coefficient, SE = standard error, CI = confidence interval, * p < 0.05, ** p < 0.01, *** p < .001

Model formula: PHQ-9 ~ psychoeducation × week + CBT exercises × week + guided sessions × week + conversations × week + baseline PHQ-9 + (1|subject)

Parameter	b	β	SE	95% CI	p
(intercept)	8.040	-	0.150	[7.745, 8.335]	0.000e+00***
conversations	6.196E-04	0.001	0.015	[-0.029, 0.030]	0.967
week	-0.440	-0.168	0.031	[-0.500, -0.379]	7.881e-46***
conversations × week	0.004	0.015	0.003	[-0.002, 0.009]	0.238
CBT exercises	-0.049	-0.022	0.109	[-0.263, 0.165]	0.653
CBT exercises × week	-0.003	-0.003	0.022	[-0.046, 0.040]	0.901
psychoeducation	0.012	0.007	0.084	[-0.153, 0.178]	0.885
psychoeducation × week	-0.022	-0.024	0.017	[-0.055, 0.011]	0.190
guided sessions	-0.097	-0.018	0.177	[-0.444, 0.249]	0.582
guided sessions × week	-0.055	-0.021	0.035	[-0.124, 0.014]	0.119
baseline PHQ-9	0.777	0.718	0.031	[0.716, 0.838]	4.241e-137***

Supplementary Table 10. Linear mixed effects model of PHQ-9 (Patient Health Questionnaire) over time (weeks) as a function of app feature engagement (psychoeducation, CBT exercises, guided sessions, or "Let's chat" conversations). b = unstandardized coefficient, β = standardized coefficient, SE = standard error, CI = confidence interval, * $p < 0.05$, ** $p < 0.01$, *** $p < .001$

Model formula: WSAS ~ psychoeducation × week + CBT exercises × week + guided sessions × week + conversations × week + baseline WSAS + (1|subject)

Parameter	b	β	SE	95% CI	p
(intercept)	15.018	-	0.325	[14.381, 15.655]	0.000e+00***
conversations	-0.005	-0.006	0.033	[-0.069, 0.059]	0.878
week	-0.480	-0.096	0.063	[-0.604, -0.357]	2.316e-14***
conversations × week	0.005	0.011	0.006	[-0.007, 0.017]	0.428
CBT exercises	-0.048	-0.012	0.237	[-0.512, 0.416]	0.838
CBT exercises × week	0.011	0.005	0.044	[-0.077, 0.098]	0.812
psychoeducation	0.295	0.082	0.182	[-0.063, 0.652]	0.106
psychoeducation × week	0.068	0.038	0.034	[-0.001, 0.136]	0.052
guided sessions	-0.542	-0.053	0.383	[-1.294, 0.209]	0.157
guided sessions × week	-0.187	-0.037	0.071	[-0.327, -0.047]	0.009**
baseline WSAS	0.759	0.655	0.038	[0.685, 0.834]	2.678e-88***

Supplementary Table 11. Linear mixed effects model of WSAS (Work and Social Adjustment Scale) over time (weeks) as a function of app feature engagement (psychoeducation, CBT exercises, guided sessions, or "Let's chat" conversations). b = unstandardized coefficient, β = standardized coefficient, SE = standard error, CI = confidence interval, * p < 0.05, ** p < 0.01, *** p < .001

Model formula: MSQ ~ psychoeducation × week + CBT exercises × week + guided sessions × week + conversations × week + baseline MSQ + (1|subject)

**Parameter**	**b**	**β**	**SE**	**95% CI**	**p**
(intercept)	21.007	-	0.297	[20.425, 21.589]	0.000e+00***
conversations	-0.010	-0.011	0.030	[-0.068, 0.049]	0.741
week	-0.652	-0.124	0.061	[-0.771, -0.533]	4.428e-27***
conversations × week	-0.003	-0.007	0.006	[-0.015, 0.008]	0.573
CBT exercises	0.167	0.038	0.216	[-0.257, 0.590]	0.440
CBT exercises × week	0.046	0.021	0.043	[-0.038, 0.130]	0.282
psychoeducation	-0.072	-0.019	0.167	[-0.399, 0.254]	0.663
psychoeducation × week	-0.047	-0.025	0.033	[-0.112, 0.018]	0.159
guided sessions	-0.349	-0.032	0.350	[-1.036, 0.338]	0.319
guided sessions × week	-0.060	-0.011	0.069	[-0.194, 0.075]	0.386
baseline MSQ	0.851	0.737	0.033	[0.787, 0.916]	8.611e-148***

Supplementary Table 12. Linear mixed effects model of MSQ (Mini Sleep Questionnaire) over time (weeks) as a function of app feature engagement (psychoeducation, CBT exercises, guided sessions, or "Let's chat" conversations). b = unstandardized coefficient, β = standardized coefficient, SE = standard error, CI = confidence interval, * p < 0.05, ** p < 0.01, *** p < .001

Supplementary Results

Baseline characteristics – engagement subgroups

Engagement subgroups were defined as follows:

1. **“Guided sessions” subgroup**: intervention participants who completed at least one guided session (n = 94)

2. **“No guided sessions” subgroup**: intervention participants who did not engage with guided sessions but started at least one CBT activity and completed one psychoeducation lesson (n = 103)

3. **“Active control” subgroup**: participants in the control group who viewed the digital workbook for at least five minutes (higher than the median viewing time of 3.8 minutes) and viewed at least six pages, which was the point at which the first CBT activity appears in each course (n = 90).

Before comparing these three subgroups, we re-assessed whether they differed on any baseline characteristics. Compared to both the active control subgroup and the “no guided sessions” subgroup, the guided sessions subgroup had significantly lower PHQ-9 scores at baseline (“guided sessions” vs “no guided sessions”: Δ = 0.61, p = .023; vs active control: Δ = 0.95, p = .001), a stronger initial preference for apps than digital workbooks (vs “no guided sessions”: Δ = 0.33, p = .032; vs active control: Δ = 0.51, p = .002), and lower Obsessive-Compulsive Index Revised (OCI-R) scores (vs “no guided sessions”: Δ = 3.52, p = .016; vs active control: Δ = 5.05, p = .003; see **Supplementary Table 12**). We therefore controlled for these baseline differences in the subsequent analysis to prevent these confounding our subgroup comparisons

Model output tables – engagement subgroups

Model formula: GAD-7 ~ subgroup × week + usage duration + baseline PHQ-9 + baseline MSQ + baseline digital workbook preference + baseline OCI-R + baseline GAD-7 + (1|subject)

Parameter	b	β	SE	95% CI	p
(intercept)	8.972	-	0.286	[8.412, 9.532]	1.745e-216***
subgroup (guided sessions)	0.036	0.007	0.404	[-0.755, 0.827]	0.928
subgroup (no guided sessions)	-0.217	-0.040	0.392	[-0.986, 0.552]	0.581
week	-0.485	-0.220	0.052	[-0.587, -0.383]	8.982e-21***
week × subgroup (guided sessions)	-0.051	-0.019	0.072	[-0.192, 0.090]	0.480
week × subgroup (no guided sessions)	0.074	0.027	0.072	[-0.066, 0.215]	0.298
usage duration (minutes)	-0.004	-0.023	0.005	[-0.015, 0.007]	0.462
baseline PHQ-9	0.061	0.069	0.035	[-0.007, 0.129]	0.078
baseline MSQ	-0.011	-0.022	0.018	[-0.045, 0.024]	0.555
baseline preference for digital workbook vs app	-0.123	-0.044	0.084	[-0.288, 0.042]	0.143
baseline OCI-R	0.024	0.061	0.013	[-0.001, 0.049]	0.062
baseline GAD-7	0.700	0.638	0.040	[0.621, 0.778]	4.367e-68***

Supplementary Table 13. Linear mixed effects model of GAD-7 (Generalized Anxiety Disorder) over time (weeks) as a function of engagement subgroups. b = unstandardized coefficient, β = standardized coefficient, SE = standard error, CI = confidence interval, * $p < 0.05$, ** $p < 0.01$, *** $p < .001$

Model formula: PHQ-9 ~ subgroup × week + usage duration + baseline PHQ-9 + baseline MSQ + baseline digital workbook preference + baseline OCI-R + (1|subject)

**Parameter**	**b**	**β**	**SE**	**95% CI**	**p**
(intercept)	9.720	-	0.324	[9.084, 10.356]	2.637e-197***
subgroup (guided sessions)	0.096	0.015	0.459	[-0.803, 0.995]	0.834
subgroup (no guided sessions)	-0.006	-9.14E-04	0.442	[-0.873, 0.861]	0.989
week	-0.447	-0.168	0.055	[-0.554, -0.340]	2.659e-16***
week × subgroup (guided sessions)	-0.063	-0.019	0.076	[-0.211, 0.086]	0.409
week × subgroup (no guided sessions)	0.015	0.004	0.075	[-0.133, 0.162]	0.847
usage duration (minutes)	-0.002	-0.011	0.006	[-0.015, 0.010]	0.725
baseline PHQ-9	0.801	0.744	0.036	[0.729, 0.872]	6.562e-107***
baseline MSQ	-0.002	-0.003	0.021	[-0.043, 0.039]	0.922
baseline preference for digital workbook vs app	-0.119	-0.035	0.098	[-0.311, 0.073]	0.225
baseline OCI-R	0.022	0.046	0.014	[-0.006, 0.050]	0.125

Supplementary Table 14. Linear mixed effects model of PHQ-9 (Patient Health Questionnaire) over time (weeks) as a function of engagement subgroups. b = unstandardized coefficient, β = standardized coefficient, SE = standard error, CI = confidence interval, * p < 0.05, ** p < 0.01, *** p < .001

Model formula: WSAS ~ subgroup × week + usage duration + baseline PHQ-9 + baseline MSQ + baseline digital workbook preference + baseline OCI-R + baseline WSAS + (1|subject)

Parameter	b	β	SE	95% CI	p
(intercept)	16.344	-	0.640	[15.090, 17.599]	7.053e-144***
subgroup (guided sessions)	0.499	0.041	0.906	[-1.278, 2.275]	0.582
subgroup (no guided sessions)	0.176	0.014	0.873	[-1.535, 1.886]	0.840
week	-0.266	-0.054	0.107	[-0.476, -0.056]	0.013*
week × subgroup (guided sessions)	-0.365	-0.060	0.149	[-0.656, -0.073]	0.014*
week × subgroup (no guided sessions)	0.135	0.022	0.148	[-0.154, 0.424]	0.360
usage duration (minutes)	0.002	0.004	0.013	[-0.023, 0.026]	0.903
baseline PHQ-9	0.514	0.258	0.081	[0.355, 0.673]	2.318e-10***
baseline MSQ	-0.116	-0.107	0.042	[-0.197, -0.034]	0.005**
baseline preference for digital workbook vs app	0.111	0.018	0.194	[-0.269, 0.491]	0.568
baseline OCI-R	0.046	0.052	0.028	[-0.010, 0.102]	0.108
baseline WSAS	0.685	0.594	0.044	[0.598, 0.772]	1.546e-53***

Supplementary Table 15. Linear mixed effects model of WSAS (Work and Social Adjustment Scale) over time (weeks) as a function of engagement subgroups. b = unstandardized coefficient, β = standardized coefficient, SE = standard error, CI = confidence interval, * p < 0.05, ** p < 0.01, *** p < .001

Model formula: MSQ ~ subgroup × week + usage duration + baseline PHQ-9 + baseline MSQ + baseline digital workbook preference + baseline OCI-R + (1|subject)

**Parameter**	**b**	**β**	**SE**	**95% CI**	**p**
(intercept)	22.172	-	0.634	[20.929, 23.414]	5.056e-268***
subgroup (guided sessions)	0.410	0.032	0.896	[-1.346, 2.167]	0.647
subgroup (no guided sessions)	0.739	0.058	0.864	[-0.955, 2.433]	0.392
week	-0.627	-0.123	0.104	[-0.831, -0.422]	1.988e-09***
week × subgroup (guided sessions)	-0.122	-0.019	0.145	[-0.406, 0.162]	0.400
week × subgroup (no guided sessions)	-0.088	-0.014	0.144	[-0.370, 0.194]	0.542
usage duration (minutes)	0.010	0.025	0.012	[-0.014, 0.035]	0.408
baseline PHQ-9	0.218	0.106	0.072	[0.078, 0.359]	0.002**
baseline MSQ	0.755	0.676	0.041	[0.675, 0.835]	1.200e-76***
baseline preference for digital workbook vs app	-0.168	-0.026	0.193	[-0.545, 0.210]	0.384
baseline OCI-R	9.763E-04	0.001	0.028	[-0.054, 0.056]	0.972

Supplementary Table 16. Linear mixed effects model of MSQ (Mini Sleep Questionnaire) over time (weeks) as a function of engagement subgroups. b = unstandardized coefficient, β = standardized coefficient, SE = standard error, CI = confidence interval, * p < 0.05, ** p < 0.01, *** p < .001

Study materials

Category	Label	Question
**Usability**	accessibility	*How well did the {tool} accommodate your needs and preferences?*
	ease of use	*How easy was it to navigate the {tool}?*
**Effectiveness**	effectiveness	*How much did the {tool} equip you with the skills to deal with mental health challenges?*
	future use	*How likely are you to use the {tool} in the future to improve your mental wellbeing?*
	learning	*How much did you apply the information learnt from the {tool} in your daily life?*
	understandability	*How much did the {tool} help you in understanding and managing your mental health?*
	usefulness	*How useful did you find the {tool} for your mental health?*
**Satisfaction**	achievement	*How much did the {tool} give you a sense of achievement?*
	motivation	*How motivated were you to use the {tool} to improve your mental health?*
	personalization	*To what degree did the {tool} provide a personalized experience?*
	satisfaction	*Overall, how satisfied are you with the {tool}?*
**Change scores**	app preference*	*Imagine that the course you completed in the {tool} was instead offered to you in the form of a {other group tool}. Which format would you prefer?*
	likelihood try therapy	*How likely are you to arrange to see a therapist in the next 3 months?*
	trust ai apps	*In general, how much do you trust wellbeing apps that use artificial intelligence (AI)?*

Supplementary Table 17. Usability and satisfaction questionnaire items. Items included in the weekly survey, plus the “change score” items that were only included in the baseline survey and the final (week 6) survey. The phrase “{tool}” was replaced by either “digital workbook” for the active control group or “app” for the intervention group. Each item was rated on a Likert scale from 1 to 5. * This item was reverse-scored for the active control group so that higher scores indicate a preference for an app over a digital workbook.

87 # Statistics for subjective ratings

Metric	Active Control	Intervention	t	95% CI	p	p_{FDR}	d
**Accessibility**	M = 2.96<br>SD = 1.27	M = 3.16<br>SD = 1.20	4.19	[0.108, 0.297]	2.830e-05***	1.038e-04***	0.16
**Ease of use**	M = 4.01<br>SD = 1.03	M = 4.37<br>SD = 0.80	10.25	[0.292, 0.431]	3.414e-24***	1.877e-23***	0.40
**Effectiveness**	M = 3.06<br>SD = 1.21	M = 3.00<br>SD = 1.22	-1.35	[-0.158, 0.029]	0.178	0.327	-0.05
**Future use**	M = 3.08<br>SD = 1.34	M = 3.14<br>SD = 1.31	1.14	[-0.042, 0.161]	0.253	0.382	0.04
**Learning**	M = 2.96<br>SD = 1.21	M = 3.01<br>SD = 1.21	1.09	[-0.042, 0.145]	0.278	0.382	0.04
**Understandability**	M = 3.24<br>SD = 1.20	M = 3.21<br>SD = 1.19	-0.49	[-0.115, 0.069]	0.627	0.689	-0.02
**Usefulness**	M = 3.23<br>SD = 1.23	M = 3.24<br>SD = 1.23	0.05	[-0.092, 0.097]	0.959	0.959	0.00
**Achievement**	M = 2.89<br>SD = 1.31	M = 2.92<br>SD = 1.32	0.57	[-0.072, 0.131]	0.566	0.689	0.02
**Motivation**	M = 2.79<br>SD = 1.35	M = 2.93<br>SD = 1.28	2.83	[0.045, 0.246]	0.005**	0.013*	0.11
**Personalization**	M = 2.57<br>SD = 1.25	M = 3.28<br>SD = 1.21	14.77	[0.618, 0.807]	1.638e-47***	1.801e-46***	0.56
**Satisfaction**	M = 3.31<br>SD = 1.13	M = 3.39<br>SD = 1.11	1.94	[-0.001, 0.171]	0.053	0.117	0.08

88 **Supplementary Table 18. Independent samples t-tests between groups (active control vs intervention) on**
89 **different questionnaire items for usability, effectiveness, and satisfaction.** M = mean, SD = standard deviation,
90 CI = confidence interval, d = Cohen's d, FDR = False Discovery Rate, * $p < 0.05$, ** $p < 0.01$, *** $p < .001$

Model formula: [subjective rating] ~ group + baseline PHQ-9 + baseline GAD-7 + comfort with technology + (1|subject)

Subjective rating	Parameter	b	β	SE	95% CI	p	p_{bonf}
Trust in mental health apps that use AI	(intercept)	2.487	-	0.058	[2.374, 2.600]	0.000e+00***	0.000e+00***
	group	0.048	0.024	0.092	[-0.132, 0.227]	0.603	1
	week	0.431	0.225	0.058	[0.316, 0.545]	1.598e-13***	4.793e-13***
	group × week	-0.408	-0.208	0.093	[-0.590, -0.226]	1.147e-05***	3.440e-05***
Preference towards using apps over digital workbooks	(intercept)	6.513	-	0.094	[6.328, 6.698]	0.000e+00***	0.000e+00***
	group	-0.013	-0.004	0.150	[-0.308, 0.281]	0.930	1
	week	0.487	0.157	0.116	[0.260, 0.715]	2.678e-05***	8.033e-05***
	group × week	-0.117	-0.037	0.185	[-0.479, 0.245]	0.527	1
Likelihood of trying therapy in the future	(intercept)	1.408	-	0.055	[1.300, 1.516]	1.709e-143***	5.128e-143***
	group	-0.006	-0.003	0.088	[-0.178, 0.166]	0.946	1
	week	0.536	0.286	0.064	[0.410, 0.661]	6.661e-17***	1.998e-16***
	group × week	-0.041	-0.022	0.102	[-0.241, 0.159]	0.686	1

Supplementary Table 19. Linear mixed-effects regression models of different questionnaire items taken at baseline and again in the final week 6 of the study. Parameter estimates (b = unstandardized, β = standardized) for the group × week interaction on each outcome metric are in bold and included in the FDR correction for multiple comparisons. β = coefficient, SE = standard error, CI = confidence interval, p_{bonf} = Bonferroni-corrected p values for 3 comparisons, * $p < 0.05$, ** $p < 0.01$, *** $p < .001$

Supplementary figures

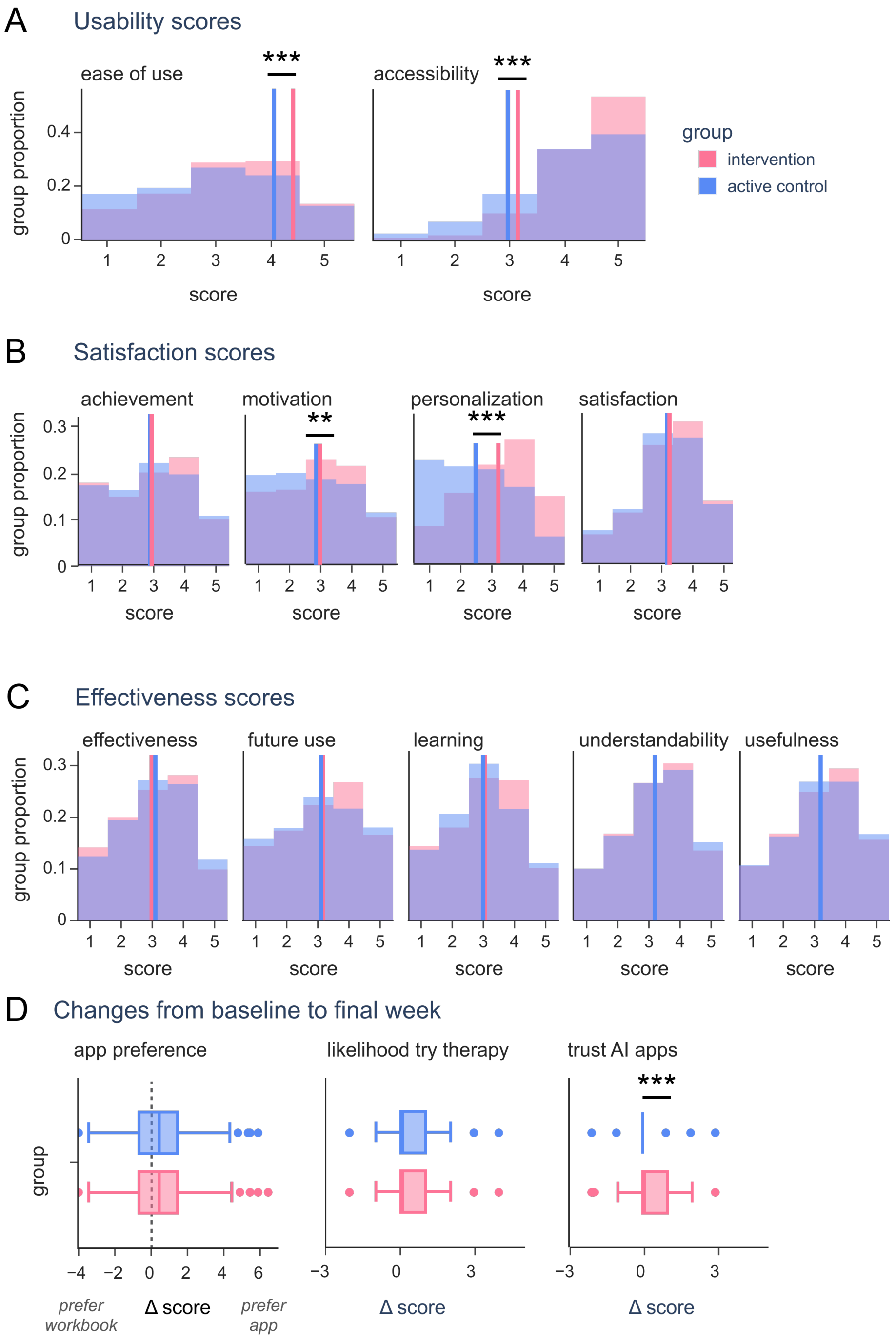

Supplementary Figure 1. Ratings of effectiveness, satisfaction, and usability. (A) Ratings for each usability survey item across the 6 weekly surveys per group (active control, n = 209: blue; intervention, n = 322: pink). Histograms represent frequency counts per score, per group, as a proportion of the total group size. Vertical lines indicate the mean score per group. **(B)** Same as **A** but for satisfaction items. *** $p < .001$ **(C)** Same as **A** but for effectiveness items. **(D)** Box plots of the change per group from baseline to the final week 6 survey across different survey items (active control: n = 174, intervention: n = 267).

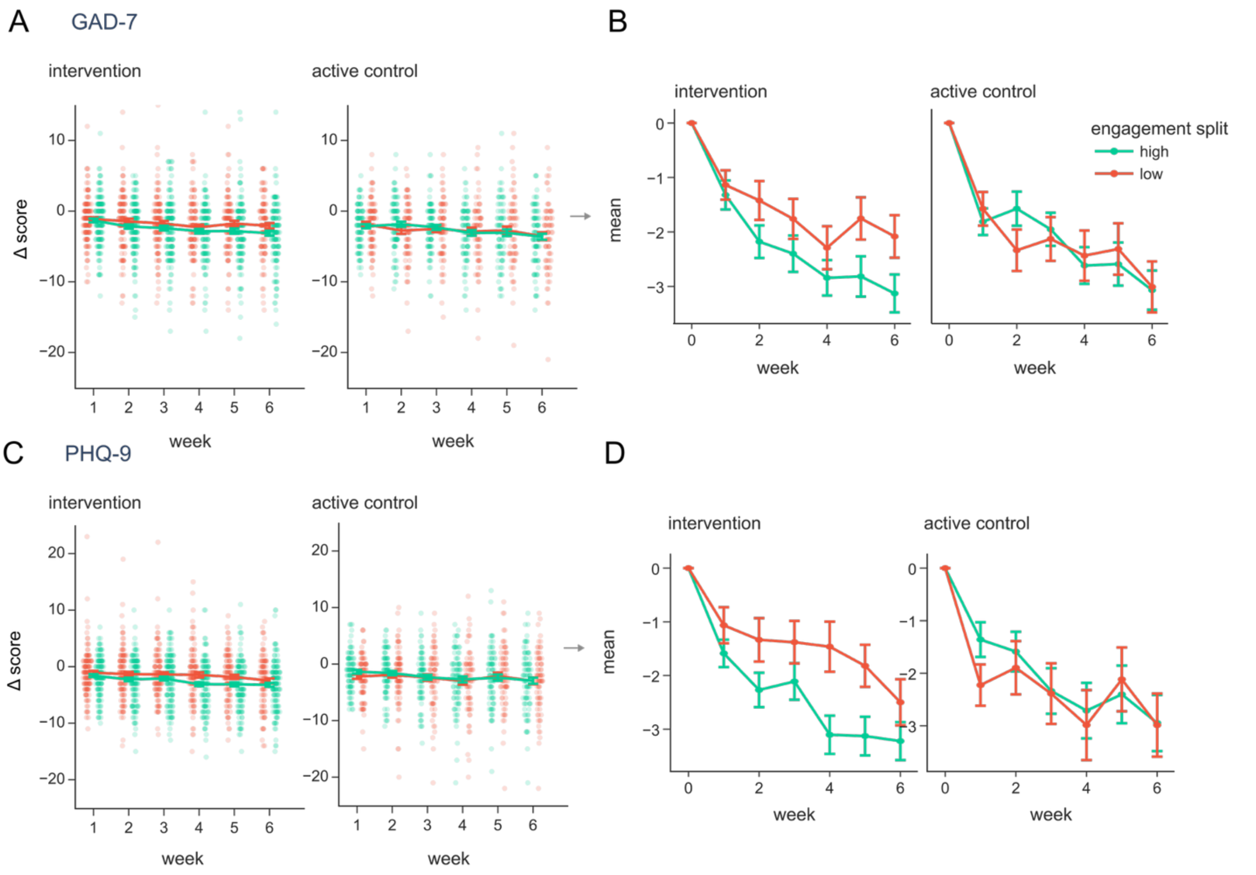

Supplementary Figure 2. Linear mixed effects regression models of symptom reduction for low and high engagement duration. (A) GAD-7 scores (y axis) per participant, per week (x axis) for the intervention (left) and control (right) groups, sub-grouped into high (green) and low (red) total engagement duration according using a median split per group (intervention and control). Lines indicate the sub-group averages per week. **(B)** Zoomed in view of the sub-group GAD-7 means per week in **A**, for intervention (left) and active control (right) groups. Error bars represent standard error of the mean. **(C)** Same as **A**, except for PHQ-9. **(D)** Zoomed in view of **C**, as in **B.**
