# Supplementary material for: Increasing engagement with cognitive-behavioral therapy (CBT) using generative AI: a randomized controlled trial (RCT)
Source: Commun Med (Lond). 2026 Jan 15;6:129. doi: 10.1038/s43856-025-01321-8 (PMC12953620; doi:10.1038/s43856-025-01321-8)
Supplement: Supplementary file 2 — Description of Additional Supplementary files [file 43856_2025_1321_MOESM2_ESM.pdf]

Description of Additional Supplementary Files

Supplementary Data 1- Baseline characteristics of each group. Continuous and categorical variables measured at baseline for the intervention and control groups. The mean (M) and standard deviation (SD) is shown for continuous variables. The number and proportion of participants included in each category is shown for binary and categorical variables (note that only one response is shown for binary variables).

Supplementary Data 2- Baseline characteristics comparison. Comparisons between different continuous and categorical variables between the active control, "no guided session", and "guided session" engagement subgroups at baseline. The mean (M) and standard deviation (SD) is shown for continuous variables, compared with independent groups t-tests. The number and proportion of participants included in each category is shown for binary and categorical variables, compared using contingency χ 2 tests across all categories (note that only one response is shown for binary variables).

Supplementary Data 3- All data generated or analysed during this study
